# Validating Analytical Protocols to Determine Selected Pesticides and PCBs Using Routine Samples

**DOI:** 10.1155/2017/9796457

**Published:** 2017-10-29

**Authors:** Oscar Pindado Jiménez, Susana García Alonso, Rosa María Pérez Pastor

**Affiliations:** Chemistry Division, CIEMAT, Av. Complutense 40, 28040 Madrid, Spain

## Abstract

This study aims at providing recommendations concerning the validation of analytical protocols by using routine samples. It is intended to provide a case-study on how to validate the analytical methods in different environmental matrices. In order to analyze the selected compounds (pesticides and polychlorinated biphenyls) in two different environmental matrices, the current work has performed and validated two analytical procedures by GC-MS. A description is given of the validation of the two protocols by the analysis of more than 30 samples of water and sediments collected along nine months. The present work also scopes the uncertainty associated with both analytical protocols. In detail, uncertainty of water sample was performed through a conventional approach. However, for the sediments matrices, the estimation of proportional/constant bias is also included due to its inhomogeneity. Results for the sediment matrix are reliable, showing a range 25–35% of analytical variability associated with intermediate conditions. The analytical methodology for the water matrix determines the selected compounds with acceptable recoveries and the combined uncertainty ranges between 20 and 30%. Analyzing routine samples is rarely applied to assess trueness of novel analytical methods and up to now this methodology was not focused on organochlorine compounds in environmental matrices.

## 1. Introduction

Organochlorine pesticides (OC) and polychlorinated biphenyls (PCBs) are of special interest because of their persistence and bioaccumulation in the environment [1–4]. This results in having been categorized as persistent organic pollutant compounds since 1995 by United Nations Environment Programme. Concern over pesticides and PCBs in environment has increased during last decades. Consequently, several studies have been conducted to increase the knowledge of the presence and effects related to these compounds [5–8]. At the beginning, many of the published works had the aim of quantifying as many pollutants as possible. Nowadays, studies are more concise, for example, about determination of pesticides residues from water [9], the occurrence of PCBs in sediments [10], transport of pesticides along a river [11, 12], or its presence in coastal areas [13].

There is an increasing demand for new analytical methods for monitoring and quantifying pesticides and PCBs at trace levels. Due to the complexity of environmental matrices and the low concentrations of these compounds, determination of pesticides and PCBs usually carried out many steps, such as concentration, separation, or extraction processes, followed by gas chromatography-mass spectrometry detection [14–16]. Therefore, possibility of losses or specific contamination often occurs. Conventional methods, such as Soxhlet extraction or liquid-liquid extraction, can be improved to adjust to matrices characteristics. It is important that the developed methods should be simple, fast, efficient, and reliable. The new chemical analysis must focus on reducing sample size, solvent volumes, and cost of analysis. It is necessary to highlight that developed methods must be always validated and their reliability must be also proved. It is well known that determination of organic compounds into interest environmental matrices is complex and shows a high variability. Some studies have established variability around 30–50% when these compounds are analyzed at trace level [17–19]. Nevertheless, the numbers of studies that have evaluated the analytical uncertainty are still less than desired [4, 20–23].

Within the framework of CIEMAT research, an analytical methodology might be performed in order to quantify pesticides and PCBs in environmental samples including a wide range of concentrations. The aims of this study were, on the one hand, developing two analytical methods to determine the selected pesticides and PCBs as fast, robust, and simple as possible in waters and sediments and, on the other hand, demonstrate the reliability of those methods by means of the analysis of routine samples. It is necessary to highlight that the present work is also intended to provide an easy to follow case-study on how to validate analytical protocols in different environmental matrices using routine samples.

Development and application of analytical methods for determination of organochlorine compounds in several matrices have already been published [9, 17, 24, 25]. Taking into account the inherent characteristics of each matrix, the estimation of possible constant/proportional bias should be also carried out to check the quality of analytical results. There studies aim to formulate mathematical algorithms to arrive at full validation, but sometimes they are difficult to apply [26].

There is still a lack of information on the variability associated with concentration level, matrix of samples, or analysis dates. To our knowledge, this approach is rarely applied for environmental studies and has not yet been done to the determination of organochlorine compounds in sediments and waters. The present work can be considered to be of the highest relevance in order to evaluate quality of any analytical method.

## 2. Materials and Methods

### 2.1. Reagents and Materials

Pesticides standard were mixture of 8 pesticides (pesticide mix-5 of 10 ng *µ*L^−1^ in cyclohexane), mixture of 4 pesticides (pesticide Mix 7 of 10 ng *µ*L^−1^ in cyclohexane), mixture of 6 pesticides (pesticide Mix 31 of 10 ng *µ*L^−1^ in acetone), mixture of 26 pesticides and PCBs (pesticide Mix 33 of 10 ng *µ*L^−1^ in isooctane), and pentachlorobenzene and pentachlorophenol (10 ng *µ*L^−1^ in cyclohexane) from Dr. Ehrenstorfer GmbH (Augsburg, Germany). Anthracene deuterated (10 g *µ*L^−1^ in cyclohexane) from Dr. Ehrenstorfer GmbH (Augsburg, Germany) and* o*-terphenyl from Supelco (Bellefonte, USA) were used as surrogate and internal standard, respectively. Two certified reference materials were used: CNS391 (CRM) of pesticides and PCBs in sediment of natural water and WatRTM pollution organochlorine pesticides 709 (ERA). Acetonitrile, dichloromethane, ethyl acetate, and hexane of residue analysis grade were supplied by J. T. Baker (Deventer, Holland). Sodium sulfate anhydrous was obtained from Merck (Darmstadt, Germany).

The SPE procedure was performed using Oasis HLB Extraction Cartridge (6 cc and 500 mg) acquired to Waters (Massachusetts, USA) in a vacuum manifold Agilent (Santa Clara, USA).

### 2.2. Instruments

An Agilent 6890 chromatograph with automatic sampler and a split/splitless programmed temperature injector coupled to an Agilent 5975 mass spectrometer was employed. Operating conditions were carrier gas: helium; capillary column HP-5MS (30 m × 0.25 mm × 0.25 *µ*m) with flow rate of 1 mL min^−1^; PTV injector with initial temperature 110°C, held for 0.5 min and then increased at a rate of 500°C min^−1^ to 200°C; operation mode splitless with 3 row injections of 1 *µ*L; initial oven temperature 100°C, held for 4 min, increased at a rate of 8°C min^−1^ to 250°C, held for 1 min, and finally increased at a rate of 8°C min^−1^ to 290°C; and detector operated in electronic impact mode (70 eV) in SIM mode.

### 2.3. Sampling

To evaluate the level of selected pesticides and PCBs, during the time period among March 2015 to October 2015, weekly samples of sediment and water were collected from a confined area of continental waters in which there were industrial discharges.

Water samples were collected in 1-liter glass bottles and stored at 4°C until analysis. 100 g of sediment samples was collected in amber glass bottles and stored at 4°C until analysis, without any additional treatment.

### 2.4. Description of Analytical Method

#### 2.4.1. Water Samples

**Figure 1:**
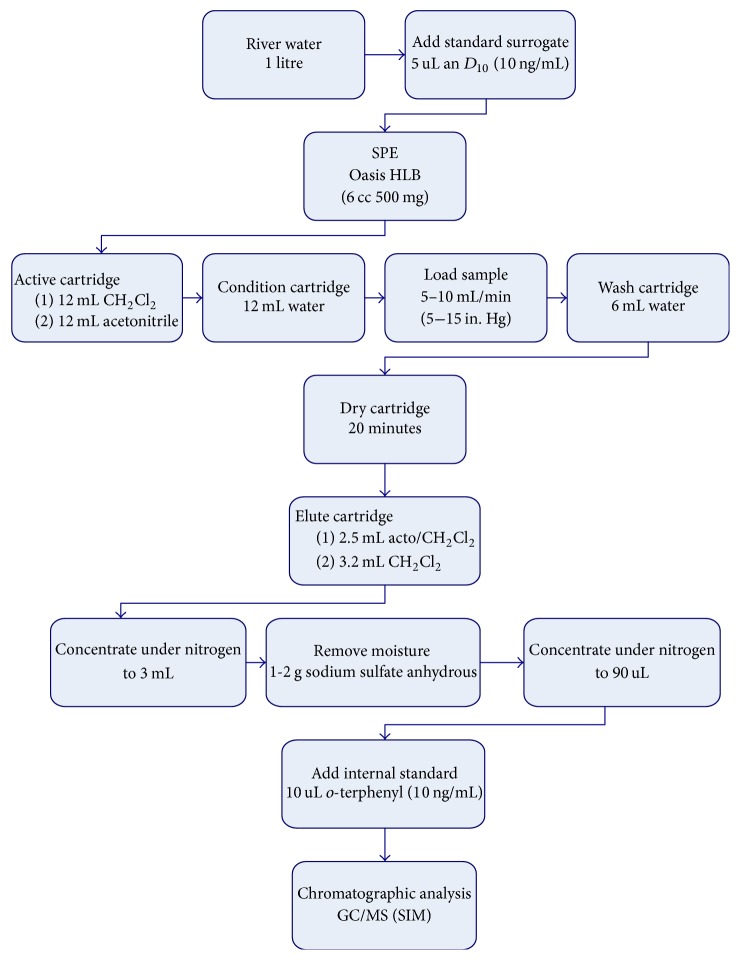
Scheme of the proposed analytical method to quantify the selected organochlorine pesticides in water.

Figure 1 shows a scheme of the proposed methodology to determine selected compounds in a water matrix. In this method, 1 L of continental water, spiked with 5 *µ*L of anthracene deuterated (10 ng *µ*L^−1^) as surrogate, is employed to determine the selected organochlorine pesticides (p,p′-DDD, o,p′-DDD, p,p′-DDE, o,p′-DDE, p,p′-DDT, o,p′-DDT, *α*-HCH, *β*-HCH, *γ*-HCH, atrazine, simazine, metolachlor, terbuthylazine, aldrin, dieldrin, endrin, isodrin, and hexachlorobenzene). Firstly, cartridges were conditioned with 12 mL of dichloromethane and 12 mL of acetonitrile and later were rinsed with 12 mL of water. Secondly, when cartridges are still wet, water sample is passed through them at a flow rate of 5–10 mL min^−1^. Cartridges were washed with 6 mL of water and then dried for 20 min under a stream of nitrogen. Later, extracts were eluted with 2.5 mL of acetonitrile/dichloromethane (1 : 1) and 3.2 mL of dichloromethane. Then, extracts were concentrated with nitrogen to 3 mL and 1 g of anhydrous sodium sulfate was added to take out humidity. Finally, extracts were concentrated with nitrogen to 90 *µ*L of ethyl acetate and 10 *µ*L of internal standard solution (10 ng *µ*L^−1^ of* o*-terphenyl in hexane) was added and injected into the GC-MS.

#### 2.4.2. Sediment Samples

**Figure 2:**
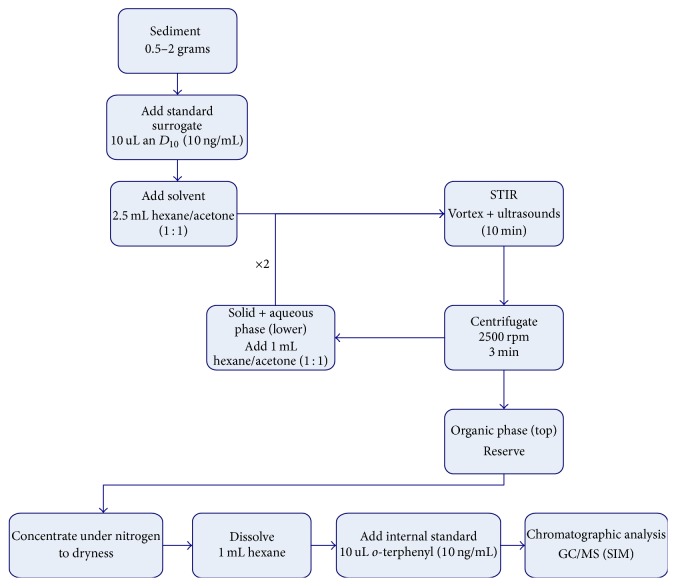
Scheme of the proposed analytical method to quantify the selected organochlorine pesticides and PCBs in sediments.

Figure 2 shows a scheme of the proposed methodology to analyze sediments matrix. The method was optimized for determining the selected organic pesticides and PCBs (p,p′-DDD, o,p′-DDD, p,p′-DDE, o,p′-DDE, p,p′-DDT, o,p′-DDT, *α*-HCH, *β*-HCH, *γ*-HCH, hexachlorobenzene, pentachlorobenzene, PCB-28, PCB-52; PCB-101; PCB-118, PCB-138, PCB-153, and PCB-180). The method begins as follows: 0.5–2 g of sediment, spiked with 10 *µ*L of anthracene deuterated (10 ng *µ*L^−1^) as surrogate, was weighed and 2.5 mL of hexane/acetone mixture was subsequently added. The first extraction was performed by shaking the mixture for 1 min in a Vortex and later goes toward an ultrasonic bath for 10 minutes. The extracts were centrifuged for 3 minutes and two phases were obtained. The top phase contains organic phase and the bottom phase (aqueous phase and sediment) was extracted twice again with the mixture hexane/acetone. All organic phases were getting together and the final extract was concentrated with nitrogen to 1 mL of hexane and 100 *µ*L of internal standard solution (10 ng *µ*L^−1^ of* o*-terphenyl in hexane) was added and injected into the GC-MS.

### 2.5. Preparation of Spiked Sediment Samples

Two sediments samples, containing a low chlorinated mass fraction, were chosen to prepare spiked samples. The spiking was conducted by adding a specific volume of chlorinated standard solutions to the sediments. In detail, volumes of 50 and 200 *µ*L of standard solutions were added to 1 gram of sediments. Spiked subsamples were agitated for a short time using automatic shaker and left 1 h for solvent evaporation. Trials of spiking on each sample were conducted according to the flow diagram presented in Figure 1S (in Supplementary Material available online at https://doi.org/10.1155/2017/9796457).

### 2.6. Mathematical Concepts Related to Constant and Proportional Bias

According to Maroto et al. [27–29], the analytical trueness can be assessed by recovery trials using routine samples and analyses of different sample masses. Taking into account that the results of sediment analysis vary within a wide range of mass fraction, the proportional and constant bias must be addressed.

#### 2.6.1. Proportional Bias

If the bias of analytical procedure varies with concentration, it can be assumed there is proportional bias. It can be estimated performing recovery measurements with spiked samples. The proportional bias is calculated from the recovery by (1). Specifically, the present work has performed two spiked levels (50 and 200 ng).

(1) $$\begin{array}{c} R_{i}=\frac{w_{found\left(i\right)}}{w_{added\left(i\right)}}, \end{array}$$

where *w*_found(*i*)_ refers to the mass fraction calculated by subtracting the mass fraction of selected organochlorine compounds measured in the spiked subsamples from that in unspiked subsample. The added mass fraction is denoted as *w*_added(*i*)_.

A significance test is then used to determine whether the mean overall recovery (*R*) is significantly different from 1.0. That is calculated as the average of *R*_*i*_ related to the two spiked levels. The test is also used to verify if the ratio between the value of *R* and its uncertainty *u*(*R*) is significant higher than the corresponding tabulated two-side value. The test statistic (*t*) was deduced according to

(2) $$\begin{array}{c} t_{R}=\frac{\left|R-1\right|}{u\left(R\right)}, \end{array}$$

where *u*(*R*) is calculated as a combined relative standard deviation *s*_*r*_(*i*) at each mass fraction level (spiked samples at low level and at high level) and considering also the corresponding results from analyses of reference material according to

(3) $$\begin{array}{c} u_{Rmean}=\sqrt{s_{r}^{2}\left(ref\;\;mat\right)+s_{r}^{2}\left(LSpk\;\;A\right)+s_{r}^{2}\left(LSpk\;\;B\right)+s_{r}^{2}\left(HSpk\;\;A\right)+s_{r}^{2}\left(HSpk\;\;B\right)}. \end{array}$$

*s*_*r*_(*i*) is calculated from the independent measurements corresponding to each subsample mass and divided by the square root of number of repetitions (*n* = 4 for reference material and *n* = 2 for each investigated mass fraction level of samples *A* and *B*).

On the basis of the study performed along this paper, the tabulated student statistic at a probability *α* = 0.05 and 11 degrees of freedom is *t*_tab_ = 2.20.

#### 2.6.2. Constant Bias

The constant bias of the analytical procedure can be evaluated by analyzing two different masses of samples and checking if both samples deliver the same result. It is estimated with the Youden method [29, 30] according to (4):

(4) xibcteai=xjbcteaj,bcte=aixj−ajxiai−aj,

where *b*_cte_ denotes the constant bias (expressed in ng), *a*_*i*_ and *a*_*j*_ are the mean values of the two masses of the subsamples for comparison, and *x*_*i*_ and *x*_*j*_ are the mean masses of quantified chlorinated compounds (ng).

To assess whether the constant bias is significant, its uncertainty must be obtained according to (5). More details can be seen in a previous work already published [27].

(5) $$\begin{array}{c} u_{r}\left(\;b_{cte}\;\right)=\frac{1}{\left(a_{i}-a_{j}\right)}\sqrt{{\left(a_{j}\frac{u\left(x_{i}\right)}{x_{i}}\right)}^{2}+{\left(a_{i}\frac{u\left(x_{j}\right)}{x_{j}}\right)}^{2}}. \end{array}$$

Similar to the above described, the significant test must be used to evaluate the constant bias by applying

(6) $$\begin{array}{c} t_{i}=\frac{b_{cte}/x_{i}}{u_{r}\left(b_{cte}\right)}. \end{array}$$

The present work has evaluated three different masses, 0.6, 1.6, and 2.6 grams of sediments, comparing them in pairs. The tabulated student statistic at a probability *α* = 0.05 and 4 degrees of freedom is *t*_tab_ = 2.78.

## 3. Results and Discussions

### 3.1. Validation of Analytical Procedures

During a time period of nine months, the linearity of the GC-MS device was examined over the range 50–1000 ng L^−1^, which is in line with the environmental levels for pesticides currently found in the literature. The calculation of a four-point linear plot establishes several linear regression and squared correlation coefficient. The GC-MS method showed good linearity with regression correlation coefficients *r*^2^ > 0.990 for all compounds along the studied period. In case of o,p′-DDT, p,p′-DDT, and several PCBs linearity was slightly lower. The authors have associated it with generation of active points into the liner through repeated injections. These compounds would be degraded, so that linearity can be affected. More details can see in the supplementary material (Table S3).

Detection limits for each compound were calculated as three time the standard deviation of four spiked samples at a final concentration of 10 ng L^−1^. The LOD and LOQ are summarized in Table S3 of supplementary material. The limits were calculated according to IUPAC recommendation, and the obtained values were below the first calibration level.

Accuracy was evaluated by analyzing two certified reference materials: ERA 709 of several pesticides in water and CNS391 of several pesticides and PCBs in sediments. Analyzed compounds showed values in the performance acceptance limits at 95% confidence interval, so accuracy is demonstrated. Results are summarized in the supplementary material (Table S4).

### 3.2. Assessing Trueness: Study of Recovery and Bias

#### 3.2.1. Water Matrices

In order to perform the recovery study, six uncontaminated water samples (500 mL) were spiked with the pesticide standards at three fortification levels (250, 500, and 1000 ng L^−1^). The lower and higher levels were spiked with standards Mix 5, Mix 7, and Mix 3, and the intermediate level was spiked with the standard Mix 33. Each fortification level was analyzed along three different weeks. The aim was to highlight how to vary the analytical response along the time. Mean recovery data obtained are summarized in Table 1.

Along those three weeks, the GC/MS device was in ideal conditions at the beginning and proceeds to nonideal conditions with time. It is noticeable that recoveries obtained in that period ranged from 40 to 120% with a relative standard deviation below 20% for the majority of compounds. On the basis of those results, the variation of analytical response with time affects mainly isomers of DDT. This fact can be explained thought generation of active points into injection device. This compound could be degraded into injection system of the gas chromatograph. In the case of recoveries below 40%, determination of these compounds must be modified.

#### 3.2.2. Sediment Matrices

The sediment samples are more complex and less homogeneous than water samples, so the recovery study must be performed in more detail. Hence, the proportional and constant biases are evaluated by employing routine samples, spiked samples, and also reference materials.

Two samples with low mass fraction are selected in order to evaluate the proportional bias [4, 27–30]. The routine samples are divided into three subsamples. One subsample is not spiked, while the other two are spiked at two different levels, as was described in the preparation of spiked sediment samples section (Figure S1). Also the bias study is carried out on two different days.

Table 2 shows the mean of recoveries, their relative standard deviation, and the statistic “*t*” parameter to determine the significance test. These parameters are obtained from the three measurement results at each mass fraction level. When the value of *t*_exp_ is higher than the tabulated two-side value (*t*_tab_ = 2.20 at a probability of *α* = 0.05, *n* = 11), the proportional bias should be considered significant, so a correction factor (1/*R*_*i*_) might be applied to correct the analytical result.

According to the summarized values in Table 2, the recovery from p,p′-DDT, *α*-HCH, *β*-HCH, and *γ*-HCH is statistically different of 100%. In the case of p,p′-DDT, an obvious matrix effect is observed in the recovery of spiked mass fraction. While recovery values in the first sample trials were acceptable (~90%), recovery results were reduced to a poor 20% in the second sediment sample trials. These observations can be related to the high susceptibility of the compound to have degradation losses into injection system of the chromatograph. On the other hand, differences for *α*-HCH, *β*-HCH, and *γ*-HCH can be attributed to their higher volatility compared to other analyzed compounds.

The constant bias study is performed by testing a routine sample heterogeneous with a high mass fraction of organochlorine compounds. This sample is divided into three subsamples of 0.6 g, 1.6 g, and 2.6 g. The analyses are done on alternate days by triplicate. More details can be seen in the supplementary material (Tables S1-S2 and Figure S2).

Table 3 compiles the values of the constant bias (*b*_cte_), its relative standard uncertainty *u*_*r*_(*b*_cte_), and the corresponding values of *t*_*i*_. When *t*_*i*_ was below the tabulated student statistic (*t*_tab_ = 2.78 at a probability *α* = 0.05 and 4 degrees of freedom), no significant constant bias is found. However, if the value of *t*_*i*_ is above that tabulated value, there is a significant constant bias.

As can be seen in Table 3, significant constant bias is obtained for p,p′-DDD, p,p′-DDE, *α*-HCH, *γ*-HCH, PCB-138, PCB-153, and PCB-180 when the analyzed subsample is lower than 1 gram. However, if the mass of the analyzed subsample is greater than 1 gram, the constant bias is insignificant, with the exception of *α*-HCH due to his higher volatility. Therefore, it can be concluded that analyzing subsamples lower than 1 gram implies significant constant bias in results. So, it is recommended to analyze subsamples higher than 2 grams. This mass is the best choice when this heterogeneous sediment is analyzed.

### 3.3. Estimation of Uncertainty

There are many sources of uncertainty associated with an analytical measurement. However, not all the components have a significant contribution to it. The cause and effect diagram facilitates identification of each contribution. The electronic supplementary material (Figures S3-S4) shows it and each mathematical equation. In this study, we have identified three main contributions to uncertainty: the concentration measured through a GC/MS calibration, the recovery study, and the intermediate precision. The remainder contributions have been already estimated elsewhere [20, 31, 32] and showed low values so those were omitted in this study.

#### 3.3.1. Concentration Measured through Calibration

This contribution (*u*_*x*_) is affected by the preparation of each standard (*u*_*C*std_) and also by the chromatographic calibrates (*u*_*Cx*_) from linear least squares.

Uncertainty associated with preparation of each calibration solution includes the uncertainty of the stock solution, according to certificates, and the uncertainty of dilution chain. The uncertainty associated with stock solution is supplied by the manufacturer as ±1%. Contributions of dilution chain include repeatability, temperature, and specification limits of syringes. According to previous studies, contributions of dilution chain were not significant. As a result, the relative uncertainty associated with each concentration of calibration standard (*u*_*C*std_/*C*std) was estimated as 0.02 (1% supplied by Dr. Ehrenstorfer, 50 *µ*g·mL^−1^) and was used for all standards in the studied concentration range.

The uncertainty from linear least squares calibration is mainly due to variability in the responses of the GC/MS. The uncertainty is evaluated from the standard deviations of slope and intercept in calibration line. For this, a set of four concentration levels is independently prepared and analyzed for a period of 4 months. In definitive, the relative uncertainty of the predicted analyte concentration (*u*_*Cx*_/*C*_*x*_) from linear least squares calibration is calculated applying

(7) $$\begin{array}{c} \frac{u_{Cx}^{2}}{C_{x}^{2}}=\frac{u_{y}^{2}}{{\left(y-b\right)}^{2}}+\frac{u_{b}^{2}}{{\left(y-b\right)}^{2}}+\frac{u_{m}^{2}}{m^{2}}, \end{array}$$

where “*y*” is the mean value of the analytical response of four standard solutions (50 ng mL^−1^ of selected organochlorinated) analyzed for a period of 4 months, “*u*_*y*_” is uncertainty, applying the expression for the lineal regression of least squares, “*b*” is the *y*-intercept of the calibration graph, “*u*_*b*_” is the uncertainty deduced from standard deviation of *y*-intercept, “*m*” is the slope of calibration graph, and “*u*_*m*_” is the uncertainty of slope.

In the electronic supplementary material, each detail related to uncertainty associated with concentration measured through calibration by GC/MS is thoroughly described (Tables S5–S7).  Table 4 summarizes the uncertainty associated with concentration measured through calibration.

The uncertainty associated with the concentration measured through calibration (*u*_*x*_/*x*) is about 15–20% for most of the selected organochlorinated compounds. Uncertainties from the calibration graphs are clearly higher than those of standards (Table 4). It is noticeable that measurements of pentachlorobenzene, isodrin, dieldrin, endrin, and PCBs numbers 101, 118, and 138 rise to 30–50% for the whole period. These results are consequence of the occurrence with time of active points into liner which degrades several compounds and so a low analytical sensitivity for those. It is important to point out that this uncertainty study was performed on intermediate terms. So those values are a clear proof of the requirement to analyze standards and sample extracts on the same day or days very close, trying to keep the same chromatographic conditions as liner, columns state, and others.

#### 3.3.2. Recovery Study

In environmental studies, there are recovery factors different from 100%. So, recovery is a significant uncertainty source. In order to estimate this contribution (*u*_rec_), the recovery study described above was employed. For water samples, the standard relative deviation from the recovery study of uncontaminated water samples spiked with pesticides at three fortification levels was employed. Meanwhile, for sediment samples, the standard relative deviation from reference material and spiking assays were used. More details can be seen in the supplementary electronic material (Tables S8 and S9).  Table 5 summarizes the final results.

As can be seen, the influence of the matrix type in the dispersion of analytical results is more significant in the case of sediment samples, with values around 10%, while those for water samples are mainly around 5%.

#### 3.3.3. Intermediate Precision

In water samples, the intermediate precision is assessed by analyzing by duplicating a set of water samples spiked at three concentration levels for a period of three weeks. The supplementary electronic material (Table S10) shows more details. The intermediate precision expressed as pooled relative standard deviation was deduced as follows:

(8) $$\begin{array}{c} {RSD}_{pooled}=\sqrt{\frac{\sum {RSD}_{i}^{2}\left(n_{i}-1\right)}{\sum \left(n_{i}-1\right)}}. \end{array}$$

Being grouped, the relative standard deviations (RSD) of the analytical results by sample are replicated. The uncertainty associated with the intermediate precision for water samples was calculated as follows:

(9) $$\begin{array}{c} u\left(\;{RSD}_{pooled}\;\right)={rsd}_{pooled}. \end{array}$$

Table 6 summarizes the results. According to that, values are lower than 20%.

In the case of sediment samples, the intermediate precision is also calculated from the analyses of a set of 33 samples collected in the area of study, which are analyzed by duplicate for a period of 9 months. More details can be seen in the electronic supplementary material (Table S11). Results of uncertainty associated with intermediate precision are summarized in Table 7.

According to values summarized in Table 7, the intermediate precision ranges between 25 and 40% for most of the analyzed compounds. These values are in line with expected levels for the analysis of organic samples in complex and heterogeneous samples. It is noteworthy that precision is evaluated with samples collected in the study area along 9 months and are analyzed by duplicate. And thus, this study is a clear and detailed item to demonstrate variability and difficulty associated with these analyses.

Particularly, the o,p′-DDT and p,p′-DDT show the highest values of RSD. It is well known that these compounds can be degraded into injection system, causing analytical problems. Also, the determination of both compounds in the analyzed matrices is at trace level, close to detection limit and in a reduced number of samples. The authors have identified these issues as responsible for those levels. In the case of pentachlorobenzene, the value measured is due to volatility of this compound, which can generate losses along the extraction process.

#### 3.3.4. Estimation of Total Uncertainty

Once the three main uncertainty contributions are calculated, the total uncertainty can be achieved from different ways. The authors have clearly reflected the different approach to be adopted to analyze the selected compounds in different matrices. The main problems of sediments matrices are inhomogeneity and, in case of the water, low concentrations.

The total uncertainty associated with the determination of selected organochloride compounds from water samples is estimated as a combined uncertainty. The relationship between the combined standard uncertainty and the uncertainty of the independent contributions on which it depends is

(10) ucPesticideswater=urcal2+urrec2+urrsdpooled2.

**Figure 3:**
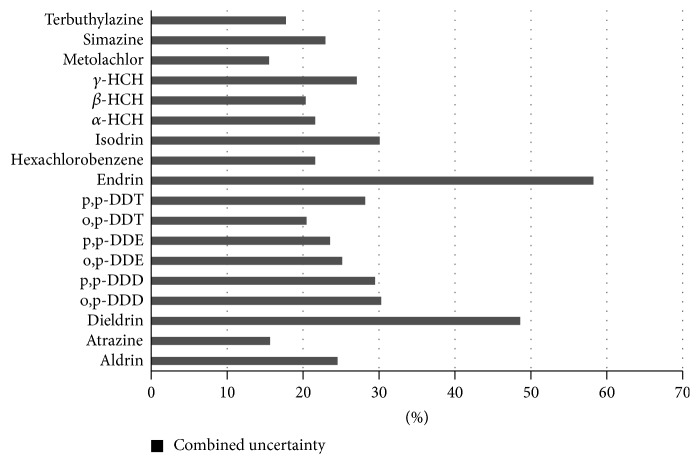
Combined uncertainty for the analysis of the selected pesticides in water samples.

Figure 3 displays the combined uncertainty for the determination of each organochlorine compound in water. As can be seen, the combined uncertainty shows values lower than 30% for many compounds. The uncertainty due to recovery has got the least influence in the final results. However, this uncertainty could be reduced somewhat. This contribution can be associated with losses of more volatile compounds during concentration under a gentle stream of nitrogen and also with incomplete retention by the SPE cartridges. If both issues are improved, the uncertainty of recovery will be slightly decreased. Secondly, the contribution of intermediate precision shows values ranged between 10 and 20%. This contribution involves variations along the time for the analysis of the spiked water samples at different levels, so robustness of methodology is clearly verified. And finally, the contribution due to the analysis shows the highest values. It would be therefore very difficult to reduce the associated uncertainty. Only, the use of new chromatographic columns, inlet systems, or more sensitive detectors will enable us to make practical improvements. In the case of the value of endrin, uncertainty is associated with well-known degradation of this compound to endrin ketone and endrin aldehyde into the inlet systems. Nevertheless, it is highlighted that the uncertainty measured involves each calibration performed along nine months. And thus, nonoptimal conditions are also considered, in order to point the need of periodic evaluation of this degradation. The authors have finally concluded that inlet system must be substituted periodically, and thus that issue does not affect the final results.

The total uncertainty associated with the determination of selected organochloride compounds from sediment samples is evaluated differently. In this case, the three main contributions cannot be combined because the three contributions are not independent.

Uncertainty contributions estimated in the present work from proportional/constant bias of sediment subsample measurements are deduced from the values above included in Tables 2 and 3. Hence, component of proportional bias is deduced as a combined value of the variances from respective assays, as it was previously indicated. Taking into account that uncertainty components associated with constant bias are estimated from assays carried out with different samples, the corresponding uncertainty contribution is estimated as an intermediate precision by means of a pooled relative standard from deduced uncertainty contributions of constant bias (*u*(*b*_cte_)%).

**Figure 4:**
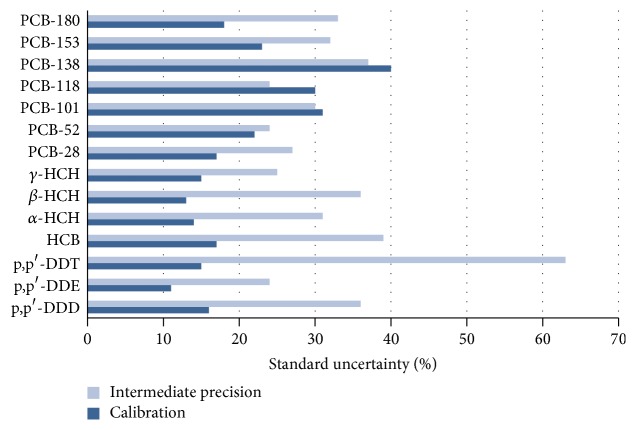
Standard uncertainty for intermediate precision and calibration contributions for the analysis of selected pesticides and PCBs in sediments.

**Figure 5:**
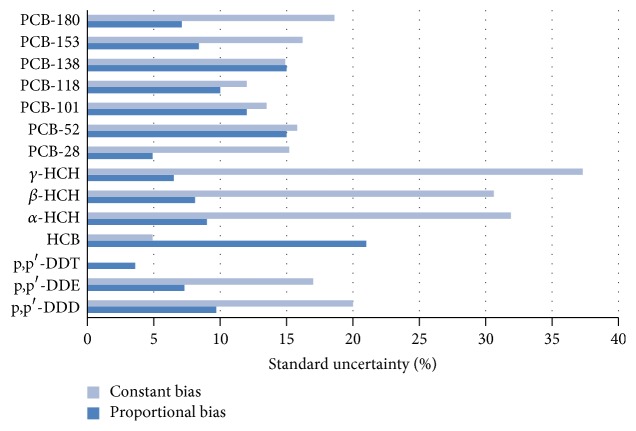
Standard uncertainty for constant and proportional bias contributions for the analysis of selected pesticides and PCBs in sediments.

Figures 4 and 5 are the representation for comparison among the uncertainties contributions deduced in this work. In general, the analytical variability from intermediate precision is the highest contribution to uncertainty. The values of intermediate precision correspond to the set of analytical measurements made during 9 months with different complexity of different sediment samples. It can be considered that those values are indicative of the total uncertainty. In general, the variability ranged between 25 and 35% for most of the studied compounds, except for p,p′-DDT and hexachlorobenzene, due to their degradation and volatility, respectively.

From the results, the variability data associated with the presence of bias indicates that the values of constant bias are higher than those of proportional bias. This effect is mainly observed for DDT, HCB, and isomers of HCH type.

## 4. Conclusions

This work describes two analytical procedures for quantifying selected organochlorine compounds in water at concentrations of ng L^−1^ and in sediments at mass fraction of mg Kg^−1^. The analytical protocols use a minimal handling and reagents for analyzing the goal compounds in sediments and waters. Both analytical methods show adequate validation parameters for quantifying those compounds at trace levels. The method for analyzing water samples involves a solid phase extraction and a GC/MS analysis; meanwhile, the method for analyzing sediments comprises a sequential mechanic and ultrasonic extractions followed by GC/MS analysis.

The novelty of this work focuses on the method validation procedure using routine samples. That assessment is rarely applied. Hence, the present work is of highest significance in order to assure quality results for monitoring organochlorine compounds in environmental matrices.

The proposed analytical method for sediments provides an important reduction of costs associated with analyses minimizing extraction time, volume of solvents, and sample weight. These aspects make an easier, cheaper, and faster method than classical extraction procedures.

In order to evaluate the performance of the proposed method, the bias and precision have been evaluated by the analysis of routine samples. One of the main objectives was to optimize the sample size, while maintaining the absence of a significant bias. The proportional bias from recovery tests is only significant in the case of p,p′-DDT and HCH isomers. Similarly, the constant bias is not significant when aliquots of 2 g were chosen. Lower weights lead to significant constant bias, which could imply revision or correction of the final results. Moreover, the evaluation of uncertainty is carried out by grouping the intermediate precision and the bias study. In general, the results are very consistent, showing a range between 25 and 35%.

The analytical methodology to determine the selected compounds in water allows quantifying them at trace levels with acceptable recoveries. Due to homogeneity of water matrices, the assessing of trueness is performed by a recovery study. Moreover, three main sources of uncertainty have been identified: recovery, calibration of GC/MS device, and intermediate precision. Finally, the combined uncertainty ranges between 20 and 30% for all compounds, with the exception of endrin. That value is due to well-known degradation of this compound into the inlet systems of a gas chromatography. We have concluded that inlet system must be substituted periodically in order to avoid errors.

Finally, the authors give case-study on how to validate and estimate uncertainty for the determination of selected compounds in function of type of matrix. This work also aimed at highlighting that validation and the evaluation of the uncertainty associated must be adapted to type of matrices, being an essential part of an environmental analysis.

## Supplementary Material

Fig. S1: Flow diagram of the trials for the calculation of proportional bias.
Fig. S2: Flow diagram of the trials for the calculation of constant bias.
Table S1: Results from analyses of two masses of sub-sample (A=0.587 g, B=1.573 g) and calculations to estimate constant bias.
Table S2: Results from analyses of two masses of sub-sample (B=1.573 g, C=2.558 g) and calculations to estimate constant bias.
Table S3: Calibration parameters and limits for selected compounds.
Table S4: Accuracy evaluated by the analysis of two certified reference materials.
Fig. S3: Cause and effect diagram.
Fig. S4: Equations to calculate uncertainty.
Table S5: Uncertainty from analytical signal (u_y_^2^).
Table S6: Uncertainty from slope (u_m_^2^) and y-intercept (u_b_^2^).
Table S7: Uncertainty associated to concentration measured through calibration.
Table S8: Uncertainty associated to recovery of selected pesticides in water samples.
Table S9: Uncertainty associated to recovery of selected pesticides in sediment samples.
Table S10: Uncertainty associated to intermediate precision for the analysis of selected compounds in water samples.

## Figures and Tables

**Table 1:** Recovery study for the analysis of six spiked water samples at three levels of concentration in different weeks.

Pesticide	Recovery (%)		
	Week 1 (500 ng L^−1^)	Week 2 (1000 ng L^−1^)	Week 3 (250 ng L^−1^)
Aldrin	59 ± 14	—	—
Atrazine	—	117 ± 14	110 ± 16
o,p′-DDD	—	60 ± 8	51 ± 8
p,p′-DDD	89 ± 23	70 ± 10	61 ± 6
o,p′-DDE	—	41 ± 4	33 ± 1
p,p′-DDE	73 ± 21	45 ± 6	34 ± 1
o,p′-DDT	—	43 ± 4	36 ± 4
p,p′-DDT	75 ± 30	68 ± 8	50 ± 9
Dieldrin	101 ± 24	88 ± 21	75 ± 10
Endrin	123 ± 21	73 ± 9	89 ± 20
Hexachlorobenzene	78 ± 20	—	—
*α*-HCH	111 ± 11	114 ± 28	117 ± 17
*β*-HCH	117 ± 12	106 ± 12	116 ± 28
*γ*-HCH	113 ± 14	112 ± 27	116 ± 23
Isodrin	64 ± 16	—	—
Metolachlor	—	91 ± 9	109 ± 11
Simazine	—	113 ± 12	120 ± 19
Terbuthylazine	—	76 ± 9	109 ± 14

**Table 2:** Values of recovery and relative standard deviations (expressed in %) obtained from the analysis of two sediments spiked at two levels of concentration.

Compound	Spiked 50 ng		Spiked 200 ng		*R*_mean_ (%)	*u*(*R*_mean_) (%)	*t*
	Sediment 1 (%)	Sediment 2 (%)	Sediment 1 (%)	Sediment 2 (%)			
p,p′-DDD	129 ± 5.7	104 ± 5.8	124 ± 2.9	82 ± 1.0	109	9.7	0.71
p,p′-DDE	103 ± 1.6	75 ± 1.2	92 ± 3.9	82 ± 5.7	88	7.3	1.62
p,p′-DDT	96 ± 1.3	26 ± 3.5	88 ± 1.6	15 ± 1.5	56	3.6	**12**
HCB	60 ± 18	122 ± 20.0	59 ± 7.0	100 ± 6.1	85	21.0	0.74
*α*-HCH	92 ± 8.8	85 ± 1.7	56 ± 5.2	87 ± 2.5	72	9.0	**3.06**
*β*-HCH	134 ± 6.3	126 ± 5.2	123 ± 6.0	126 ± 4.7	127	8.1	**3.37**
*γ*-HCH	71 ± 1.0	89 ± 5.3	67 ± 4.3	93 ± 1.0	80	6.5	**3.10**
PCB-28	108 ± 2.6	137 ± 6.0	99 ± 5.8	89 ± 16.0	108	4.9	1.64
PCB-52	112 ± 1.1	109 ± 2.9	101 ± 2.7	111 ± 7.5	108	15.0	0.54
PCB-101	120 ± 1.0	101 ± 3.3	112 ± 3.5	104 ± 4.3	109	12.0	0.77
PCB-118	113 ± 4.0	103 ± 3.6	118 ± 2.4	104 ± 1.6	110	10.2	0.93
PCB-138	120 ± 1.5	90 ± 4.6	114 ± 1.1	92 ± 1.1	104	11.9	0.35
PCB-153	120 ± 1.0	94 ± 3.7	113 ± 1.9	97 ± 1.5	106	8.4	0.71
PCB-180	129 ± 1.2	82 ± 8.3	123 ± 1.0	79 ± 1.1	103	7.1	0.48

**Table 3:** Study of constant bias by means the analysis of three sediment subsamples of 0.6, 1.6, and 2.6 grams, respectively.

(ng)	Subsample 1	Subsample 2	Subsample 3	*b*_cte_		*u*(*b*_cte_)%		*t*(*b*_cte_)	
	*x*_1=0.6_	*x*_2=1.6_	*x*_3=2.6_	*x*_1_-*x*_2_	*x*_2_-*x*_3_	*x*_1_-*x*_2_	*x*_2_-*x*_3_	*x*_1_-*x*_2_	*x*_2_-*x*_3_
o,p′-DDD	44 ± 5.2	102 ± 5.2	160 ± 28	10	−7.5	0.109	0.209	2.2	0.4
p,p′-DDD	45 ± 3.4	99 ± 12	143 ± 8.3	14	−29	0.080	0.195	**3.7**	1.5
o,p′-DDE	4.3 ± 0.26	11 ± 0.41	17 ± 3.4	0.47	−0.58	0.059	0.231	1.9	0.2
p,p′-DDE	23 ± 2.1	52 ± 1.6	78 ± 11	6.5	−8.9	0.085	0.165	**3.3**	1.0
HCB	3524 ± 209	8451 ± 198	13832 ± 421	594	154	0.055	0.049	3.1	0.4
*α*-HCH	2.9 ± 0.06	15 ± 2.6	41 ± 6.0	−4.1	27	0.066	0.319	**22**	**5.9**
*β*-HCH	61 ± 4.6	145 ± 29	261 ± 16	11	40	0.098	0.306	1.8	0.9
*γ*-HCH	8.2 ± 1.7	18 ± 1.9	40 ± 12	2.5	18	0.190	0.373	**5.8**	2.7
PCB-28	442 ± 49	997 ± 77	1607 ± 138	112	−23	0.105	0.152	2.4	0.1
PCB-52	143 ± 18	352 ± 25	576 ± 59	19	7.1	0.119	0.158	1.6	0.1
PCB-101	68 ± 5.8	178 ± 16	252 ± 2.8	2.8	−60	0.085	0.135	0.5	2.5
PCB-118	30 ± 5.0	63 ± 4.4	104 ± 5.5	11	1.5	0.154	0.120	2.3	0.2
PCB-138	3.3 ± 0.15	7.7 ± 0.59	13 ± 1.1	0.65	1.4	0.050	0.149	**3.9**	1.2
PCB-153	10 ± 0.63	22 ± 2.1	40 ± 3.0	3.0	5.2	0.065	0.162	**4.5**	1.4
PCB-180	14 ± 2.1	25 ± 2.6	38 ± 3.4	7.2	−3.8	0.144	0.186	**3.6**	0.8

**Table 4:** Uncertainty associated with concentration measured through calibration.

	(*u*_*C*std_)^2^/*C*_std_^2^	(*u*_*Cx*_)^2^/*C*_*x*_^2^	*u*_*x*_^2^/*x*^2^	*u*_*x*_/*x*
Aldrin	0.0004	0.032	0.032	0.18
Atrazine	0.0004	0.014	0.014	0.12
Dieldrin	0.0004	0.212	0.212	0.46
o,p′-DDE	0.0004	0.007	0.007	0.08
p,p′-DDE	0.0004	0.011	0.012	0.11
o,p′-DDD	0.0004	0.019	0.019	0.14
p,p′-DDD	0.0004	0.026	0.026	0.16
o,p′-DDT	0.0004	0.025	0.026	0.16
p,p′-DDT	0.0004	0.021	0.021	0.14
Endrin	0.0004	0.251	0.252	0.50
Hexachlorobenzene	0.0004	0.027	0.027	0.16
Isodrin	0.0004	0.061	0.061	0.25
*α*-HCH	0.0004	0.018	0.018	0.13
*β*-HCH	0.0004	0.015	0.016	0.13
*γ*-HCH	0.0004	0.022	0.023	0.15
Metolachlor	0.0004	0.016	0.016	0.13
Pentachlorobenzene	0.0004	0.120	0.120	0.35
PCB-28	0.0004	0.027	0.027	0.16
PCB-52	0.0004	0.050	0.050	0.22
PCB-101	0.0004	0.093	0.093	0.30
PCB-118	0.0004	0.090	0.091	0.30
PCB-138	0.0004	0.158	0.159	0.40
PCB-153	0.0004	0.053	0.053	0.23
PCB-180	0.0004	0.033	0.034	0.18
Simazine	0.0004	0.040	0.041	0.20
Terbuthylazine	0.0004	0.017	0.018	0.13

**Table 5:** Results of uncertainty associated with recovery of selected pesticides in water and sediment samples (n.a.: nonanalyzed).

	Water	Sediment
	*u*_rec_	*u*(*R*_mean_)
Aldrin	0.019	n.a.
Atrazine	0.037	n.a.
Dieldrin	0.058	n.a.
o,p-DDE	0.062	n.a.
p,p-DDE	0.086	0.089
o,p-DDD	0.070	n.a.
p,p-DDD	0.072	0.103
o,p-DDT	0.073	n.a.
p,p-DDT	0.114	0.047
Endrin	0.130	n.a.
Hexachlorobenzene	0.066	0.205
Isodrin	0.033	n.a.
*α*-HCH	0.062	0.090
*β*-HCH	0.051	0.081
*γ*-HCH	0.054	0.065
Metolachlor	0.035	n.a.
PCB-28	n.a.	0.137
PCB-52	n.a.	0.100
PCB-101	n.a.	0.113
PCB-118	n.a.	0.102
PCB-138	n.a.	0.119
PCB-153	n.a.	0.083
PCB-180	n.a.	0.087
Simazine	0.044	n.a.
Terbuthylazine	0.060	n.a.

**Table 6:** Intermediate precision calculated for determination of selected pesticides in water.

Compound	Average	*n*	rsd_pooled_	*u*(rsd_*p*_)	*u*_*r*_(rsd_*p*_) (%)
Aldrin	0.49	3	0.082	0.082	17
Atrazine	1.11	6	0.104	0.104	9
o,p′-DDD	0.66	9	0.172	0.172	15
p,p′-DDD	0.63	6	0.150	0.150	26
o,p′-DDE	0.37	6	0.085	0.085	24
p,p′-DDE	0.50	9	0.096	0.096	23
o,p′-DDT	0.60	6	0.063	0.063	19
p,p′-DDT	0.76	9	0.160	0.160	10
Dieldrin	0.81	9	0.217	0.217	21
Endrin	0.82	9	0.120	0.120	27
HCB	0.78	3	0.090	0.090	12
*α*-HCH	1.28	9	0.199	0.199	16
*β*-HCH	1.17	9	0.182	0.182	16
*γ*-HCH	1.20	9	0.176	0.176	15
Isodrin	0.54	3	0.121	0.121	22
Metolachlor	0.98	6	0.076	0.076	8
Simazine	1.17	6	0.121	0.121	10
Terbuthylazine	0.91	6	0.095	0.095	11

**Table 7:** Intermediate precision assessed by the analysis of different sediments collected in the area of study. The number of sediment samples corresponds to those of samples with mass fraction above detection limit.

Compound	Number of sediment samples	RSD_pooled_ (%)
o,p′-DDD	32	23
p,p′-DDD	32	36
o,p′-DDE	32	23
p,p′-DDE	31	24
o,p′-DDT	14	54
p,p′-DDT	10	63
Hexachlorobenzene	31	39
*α*-HCH	32	31
*β*-HCH	29	36
*γ*-HCH	27	25
Pentachlorobenzene	27	43
PCB-28	31	27
PCB-52	32	24
PCB-101	31	30
PCB-118	18	24
PCB-138	11	37
PCB-153	29	32
PCB-180	9	33

## References

[1] Guan Y.-F., Wang J.-Z., Ni H.-G., Zeng E. Y. (2009). Organochlorine pesticides and polychlorinated biphenyls in riverine runoff of the Pearl River Delta, China: Assessment of mass loading, input source and environmental fate. *Environmental Pollution*.

[2] Hong S. H., Yim U. H., Shim W. J., Oh J. R., Viet P. H., Park P. S. (2008). Persistent organochlorine residues in estuarine and marine sediments from Ha Long Bay, Hai Phong Bay, and Ba Lat Estuary, Vietnam. *Chemosphere*.

[3] Ratola N., Santos L., Herbert P., Alves A. (2006). Uncertainty associated to the analysis of organochlorine pesticides in water by solid-phase microextraction/gas chromatography-electron capture detection-Evaluation using two different approaches. *Analytica Chimica Acta*.

[4] Yenisoy-Karakaş S. (2006). Validation and uncertainty assessment of rapid extraction and clean-up methods for the determination of 16 organochlorine pesticide residues in vegetables. *Analytica Chimica Acta*.

[5] Lacorte S., Quintana J., Tauler R., Ventura F., Tovar-Sánchez A., Duarte C. M. (2009). Ultra-trace determination of Persistent Organic Pollutants in Arctic ice using stir bar sorptive extraction and gas chromatography coupled to mass spectrometry. *Journal of Chromatography A*.

[6] Castells P., Parera J., Santos F. J., Galceran M. T. (2008). Occurrence of polychlorinated naphthalenes, polychlorinated biphenyls and short-chain chlorinated paraffins in marine sediments from Barcelona (Spain). *Chemosphere*.

[7] Bustnes J. O., Miland Ø., Fjeld M., Erikstad K. E., Skaare J. U. (2005). Relationships between ecological variables and four organochlorine pollutants in an artic glaucous gull (Larus hyperboreus) population. *Environmental Pollution*.

[8] Vallack H. W., Bakker D. J., Brandt I. (1998). Controlling persistent organic pollutants-what next?. *Environmental Toxicology and Pharmacology*.

[9] Shamsipur M., Yazdanfar N., Ghambarian M. (2016). Combination of solid-phase extraction with dispersive liquid-liquid microextraction followed by GC-MS for determination of pesticide residues from water, milk, honey and fruit juice. *Food Chemistry*.

[10] Yang Z., Shen Z., Gao F., Tang Z., Niu J. (2009). Occurrence and possible sources of polychlorinated biphenyls in surface sediments from the Wuhan reach of the Yangtze River, China. *Chemosphere*.

[11] Navarro A., Tauler R., Lacorte S., Barceló D. (2010). Occurrence and transport of pesticides and alkylphenols in water samples along the Ebro River Basin. *Journal of Hydrology*.

[12] Konstantinou I. K., Hela D. G., Albanis T. A. (2006). The status of pesticide pollution in surface waters (rivers and lakes) of Greece. Part I. Review on occurrence and levels. *Environmental Pollution*.

[13] Carvalho P. N., Rodrigues P. N. R., Basto M. C. P., Vasconcelos M. T. S. D. (2009). Organochlorine pesticides levels in Portuguese coastal areas. *Chemosphere*.

[14] Yazdanfar N., Yamini Y., Ghambarian M. (2014). Homogeneous liquid-liquid microextraction for determination of organochlorine pesticides in water and fruit samples. *Chromatographia*.

[15] Cavaliere B., Monteleone M., Naccarato A., Sindona G., Tagarelli A. (2012). A solid-phase microextraction-gas chromatographic approach combined with triple quadrupole mass spectrometry for the assay of carbamate pesticides in water samples. *Journal of Chromatography A*.

[16] Jia C., Zhu X., Wang J. (2010). Extraction of pesticides in water samples using vortex-assisted liquid-liquid microextraction. *Journal of Chromatography A*.

[17] Camino-Sánchez F. J., Zafra-Gómez A., Cantarero-Malagón S., Vílchez J. L. (2012). Validation of a method for the analysis of 77 priority persistent organic pollutants in river water by stir bar sorptive extraction in compliance with the European Water Framework Directive. *Talanta*.

[18] Lepom P., Brown B., Hanke G., Loos R., Quevauviller P., Wollgast J. (2009). Needs for reliable analytical methods for monitoring chemical pollutants in surface water under the European Water Framework Directive. *Journal of Chromatography A*.

[19] Gustavo González A., Ángeles Herrador M. (2007). A practical guide to analytical method validation, including measurement uncertainty and accuracy profiles. *TrAC - Trends in Analytical Chemistry*.

[20] Pindado Jiménez Ó., Pérez Pastor R. M. (2012). Estimation of measurement uncertainty of pesticides, polychlorinated biphenyls and polyaromatic hydrocarbons in sediments by using gas chromatography-mass spectrometry. *Analytica Chimica Acta*.

[21] Drolc A., Pintar A. (2011). Measurement uncertainty evaluation and in-house method validation of the herbicide iodosulfuron-methyl-sodium in water samples by using HPLC analysis. *Accreditation and Quality Assurance*.

[22] Štěpán R., Hajšlová J., Kocourek V., Tichá J. (2004). Uncertainties of gas chromatographic measurement of troublesome pesticide residues in apples employing conventional and mass spectrometric detectors. *Analytica Chimica Acta*.

[23] Cuadros-Rodríguez L., Hernández Torres M. E., Almansa López E., Egea González F. J., Arrebola Liébanas F. J., Martínez Vidal J. L. (2002). Assessment of uncertainty in pesticide multiresidue analytical methods: Main sources and estimation. *Analytica Chimica Acta*.

[24] Souza A. S. D., Torres J. P. M., Meire R. O., Neves R. C., Couri M. S., Serejo C. S. (2008). Organochlorine pesticides (OCs) and polychlorinated biphenyls (PCBs) in sediments and crabs (Chasmagnathus granulata, Dana, 1851) from mangroves of Guanabara Bay, Rio de Janeiro State, Brazil. *Chemosphere*.

[25] Klánová J., Matykiewiczová N., Máčka Z., Prošek P., Láska K., Klán P. (2008). Persistent organic pollutants in soils and sediments from James Ross Island, Antarctica. *Environmental Pollution*.

[26] (2012). *EURACHEM. CITAC Guide CG 4. Quantifying Uncertainty in Analytical Measurement*.

[27] García-Alonso S., Pérez-Pastor R. M., Sanz-Rivera D., Rojas-García E., Rodríguez-Maroto J. (2017). PAH analysis in biomass combustion wastes: an approach to evaluate bias and precision of analytical results using routine samples. *Accreditation and Quality Assurance*.

[28] Maroto A., Boqué R., Riu J., Xavier Rius F. (2001). Estimation of measurement uncertainty by using regression techniques and spiked samples. *Analytica Chimica Acta*.

[29] Maroto A., Boqué R., Riu J., Rius F. X. (2001). Measurement uncertainty in analytical methods in which trueness is assessed from recovery assays. *Analytica Chimica Acta*.

[30] Kelly W. R., Fasset J. D. (1983). Determination of picogram quantities of uranium in biological tissues by isotope dilution thermal ionization mass spectrometry with ion counting detection. *Analytical Chemistry*.

[31] García-Alonso S., Pérez-Pastor R. M., García-Frutos F. J. (2011). An evaluation of analytical quality for selected PAH measurements in a fuel-contaminated soil. *Accreditation and Quality Assurance*.

[32] Jiménez O. P., Pastor R. M. P., Alonso S. G. (2010). Assessment uncertainty associated to the analysis of organic compounds linked to particulate matter of atmospheric aerosols. *Talanta*.

