# Supplementary material for: Validating Analytical Protocols to Determine Selected Pesticides and PCBs Using Routine Samples
Source: J Anal Methods Chem. 2017 Oct 29;2017:9796457. doi: 10.1155/2017/9796457 (PMC5676487; doi:10.1155/2017/9796457)
Supplement: Supplementary file 1 — Fig. S1: Flow diagram of the trials for the calculation of proportional bias. Fig. S2: Flow diagram of the trials for the calculation of constant bias. Table S1: Results from analyses of two masses of sub-sample (A=0.587 g, B=1.573 g) and calculations to estimate constant bias. Table S2: Results from analyses of two masses of sub-sample (B=1.573 g, C=2.558 g) and calculations to estimate constant bias. Table S3: Calibration parameters and limits for selected compounds. Table S4: Accuracy evaluated by the analysis of two certified reference materials. Fig. S3: Cause and effect diagram. Fig. S4: Equations to calculate uncertainty. Table S5: Uncertainty from analytical signal (uy2). Table S6: Uncertainty from slope (um2) and y-intercept (ub2). Table S7: Uncertainty associated to concentration measured through calibration. Table S8: Uncertainty associated to recovery of selected pesticides in water samples. Table S9: Uncertainty associated to recovery of selected pesticides in sediment samples. Table S10: Uncertainty associated to intermediate precision for the analysis of selected compounds in water samples. [file 9796457.f1.docx]

Validating analytical protocols to determine selected pesticides and PCBs using routine samples.

Oscar Pindado Jiménez, Susana García Alonso and Rosa M. Pérez Pastor.

This Electronic Supplementary Material enhances the understanding of the scientific article. This document summarise all the comments that could not be included into the text. This document is divided in 3 sections, including 4 figures and 11 tables.

Section 1: Constant and proportional bias.

Figure S1: Flow diagram of the trials for the calculation of proportional bias.

Figure S2: Flow diagram of the trials for the calculation of constant bias.

Table S1: Results from analyses of two masses of sub-samples (A=0.587 g, B= 1.573g) and calculations to estimate the constant bias.

Table S2: Results from analyses of two masses of sub-samples (B= 1.573 g, C= 2.558 g) and calculations to estimate the constant bias.

Section 2: Validation of analytical protocol.

Table S3: Calibration parameters and limits for selected compounds.

Table S4: Accuracy evaluated by the analysis of two certified reference materials.

Section 3: Uncertainty.

Figure S5: Cause and effect diagram.

Figure S6: Equations to calculate uncertainty.

[Table S5: Uncertainty from analytical signal (u_y_^2^)](#_Toc454535007).

[Table S6: Uncertainty from slope (u_m_^2^) and y-intercept (u_b_^2^)](#_Toc454535008).

[Table S7: Uncertainty associated to concentration measured through calibration](#_Toc454535009).

[Table S8: Uncertainty associated to recovery of selected pesticides in water samples](#_Toc454535010).

[Table S9: Uncertainty associated to recovery of selected pesticides in sediment samples](#_Toc454535011).

[Table S10: Uncertainty associated to intermediate precision for the analysis of selected compounds in water samples](#_Toc454535012).

[Table S11: Variance from the analysis of each sediment by duplicate.](#_Toc454535013)

Fig. S1: Flow diagram of the trials for the calculation of proportional bias.

Fig. S2: Flow diagram of the trials for the calculation of constant bias.

Table S1: Results from analyses of two masses of sub-sample (A=0.587 g, B=1.573 g) and calculations to estimate constant bias.

|  | Mass average (ng) | | Standard deviation (ng) | | Constant bias | U(x)/X | | Uncert. rel constant bias | Relative constant bias | | Statistic “t” | |
| --- | --- | --- | --- | --- | --- | --- | --- | --- | --- | --- | --- | --- |
|  | **A** | **B** | **A** | **B** | **A-B** | **A** | **B** | **A-B** | **A** | **B** | **A** | **B** |
| **α-HCH** | 2.9 | 15 | 0.1  ..14.6 | 2.6 | -4.1 | 0.013 | 0.10 | 0.066 | -1.41 | -0.28 | -22 | -4.26 |
| **β-HCH** | 6 | 145 | 4.6 | 28.9 | 11 | 0.044 | 0.11 | 0.1 | 0.18 | 0.07 | 1.82 | 0.76 |
| **γ-HCH** | 8.2 | 18 | 1.7 | 1.9 | 2.5 | 0.12 | 0.063 | 0.19 | 0.30 | 0.14 | 1.59 | 0.73 |
| **HCB** | 3524 | 8451 | 209 | 198 | 594 | 0.034 | 0.014 | 0.055 | 0.17 | 0.07 | 3.06 | 1.28 |
| **o-p’-DDE** | 4.3 | 11 | 0.3 | 0.4 | 0.5 | 0.036 | 0.022 | 0.059 | 0.11 | 0.04 | 1.86 | 0.75 |
| **p-p’-DDE** | 23. | 52 | 2.1 | 1.6 | 6.5 | 0.053 | 0.018 | 0.085 | 0.28 | 0.13 | 3.30 | 1.49 |
| **o-p’-DDD** | 44 | 102 | 5.2 | 5.2 | 10 | 0.068 | 0.029 | 0.11 | 0.24 | 0.1 | 2.16 | 0.94 |
| **p-p’-DDD** | 45 | 99 | 3.4 | 12.1 | 14 | 0.043 | 0.07 | 0.08 | 0.30 | 0.14 | 3.75 | 1.72 |
| **PCB-28** | 442 | 997 | 49 | 77 | 112 | 0.064 | 0.045 | 0.11 | 0.25 | 0.11 | 2.41 | 1.07 |
| **PCB-52** | 143 | 352 | 18 | 25 | 19 | 0.073 | 0.042 | 0.12 | 0.13 | 0.05 | 1.13 | 0.46 |
| **PCB-101** | 68 | 178 | 5.8 | 16 | 2.8 | 0.049 | 0.052 | 0.085 | 0.04 | 0.02 | 0.48 | 0.18 |
| **PCB-118** | 30 | 63 | 5.0 | 4.4 | 11 | 0.095 | 0.04 | 0.15 | 0.036 | 0.17 | 2.33 | 1.12 |
| **PCB-138** | 3.3 | 8 | 0.2 | 0.6 | 0.6 | 0.027 | 0.044 | 0.05 | 0.20 | 0.08 | 3.93 | 1.67 |
| **PCB-153** | 10 | 22 | 0.6 | 2.1 | 3.0 | 0.036 | 0.053 | 0.065 | 0.29 | 0.13 | 4.49 | 2.05 |
| **PCB-180** | 14 | 25 | 2.1 | 2.6 | 7.2 | 0.088 | 0.06 | 0.14 | 0.52 | 0.29 | 3.61 | 2.00 |

Table S2: Results from analyses of two masses of sub-sample (B=1.573 g, C=2.558 g) and calculations to estimate constant bias.

|  | Mass average (ng) | | Standard deviation (ng) | | Constant bias | U(x)/X | | Uncert. rel constant bias | Relative constant bias | | Statistic “t” | |
| --- | --- | --- | --- | --- | --- | --- | --- | --- | --- | --- | --- | --- |
|  | **B** | **C** | **B** | **C** | **B-C** | **B** | **C** | **B-C** | **B** | **C** | **B** | **C** |
| **α-HCH** | 15 | 41 | 2.6 | 6.0 | 27 | 0.10 | 0.10 | 0.32 | 1.88 | 0.67 | 5.88 | 2.10 |
| **β-HCH** | 145 | 261 | 28.9 | 15.6 | 40 | 0.11 | 0.042 | 0.31 | 0.28 | 0.15 | 0.90 | 0.50 |
| **γ-HCH** | 18 | 40 | 1.9 | 11.8 | 18 | 0.063 | 0.21 | 0.37 | 0.99 | 0.44 | 2.66 | 1.19 |
| **HCB** | 8451 | 13832 | 198 | 421 | 154 | 0.014 | 0.022 | 0.05 | 0.02 | 0.01 | 0.37 | 0.23 |
| **o-p’-DDE** | 11 | 17 | 0.4 | 3.4 | -0.6 | 0.022 | 0.14 | 0.23 | -0.05 | -0.03 | -0.23 | -0.15 |
| **p-p’-DDE** | 52 | 78 | 1.6 | 10.9 | -8.9 | 0.018 | 0.10 | 0.16 | -0.17 | -0.11 | -1.05 | -0.69 |
| **o-p’-DDD** | 102 | 160 | 5.2 | 27.6 | -7.5 | 0.029 | 0.12 | 0.21 | -0.07 | -0.05 | -0.35 | -0.22 |
| **p-p’-DDD** | 99 | 143 | 12.1 | 8.3 | -29 | 0.07 | 0.041 | 0.19 | -0.29 | -0.20 | -1.50 | -1.04 |
| **PCB-28** | 997 | 1607 | 77 | 138 | -23 | 0.045 | 0.061 | 0.15 | -0.02 | -0.01 | -0.15 | -0.09 |
| **PCB-52** | 352 | 576 | 25 | 59 | 7.1 | 0.042 | 0.072 | 0.16 | 0.02 | 0.01 | 0.13 | 0.08 |
| **PCB-101** | 178 | 252 | 16 | 2.8 | -60 | 0.052 | 0.008 | 0.14 | -0.34 | -0.24 | -2.50 | -1.77 |
| **PCB-118** | 63 | 104 | 4.4 | 5.5 | 1.5 | 0.04 | 0.038 | 0.12 | 0.02 | 0.01 | 0.20 | 0.12 |
| **PCB-138** | 8 | 13 | 0.6 | 1.1 | 1.4 | 0.044 | 0.060 | 0.15 | 0.18 | 0.1 | 1.19 | 0.69 |
| **PCB-153** | 22 | 40 | 2.1 | 3.0 | 5.2 | 0.053 | 0.053 | 0.16 | 0.23 | 0.13 | 1.43 | 0.81 |
| **PCB-180** | 25 | 38 | 2.6 | 3.4 | -3.8 | 0.06 | 0  .063 | 0.19 | -0.15 | -0.1 | -0.82 | -0.54 |

Table S3: Calibration parameters and limits for selected compounds

| Compound | Number calibrations | Range of r^2^ | Linear range  (ng L^-1^) | LOD  (ng L^-1^) | LOQ  (ng L^-1^) |
| --- | --- | --- | --- | --- | --- |
| **Aldrin** | 7 | 0.997 - 0.99997 | 50 – 1000 | 2 | 5 |
| **Atrazine** | 9 | 0.994 - 0.9997 | 50 – 2000 | 4 | 7 |
| **o-p’-DDD** | 9 | 0.991 - 0.9997 | 50 – 1000 | 3 | 21 |
| **p-p’-DDD** | 13 | 0.997 - 0.99995 | 50 – 1000 | 3 | 8 |
| **o-p’-DDE** | 9 | 0.990 - 0.9991 | 50 – 1000 | 4 | 18 |
| **p-p’-DDE** | 13 | 0.990 - 0.99993 | 50 – 1000 | 1 | 3 |
| **o-p’-DDT** | 9 | 0.990 - 0.9998 | 50 – 1000 | 2 | 4 |
| **p-p’-DDT** | 13 | 0.991 - 0.9992 | 50 – 1000 | 2 | 5 |
| **Dieldrin** | 15 | 0.991 - 0.99998 | 50 – 1000 | 3 | 5 |
| **Endrin** | 15 | 0.992 - 0.99993 | 50 – 1000 | 2 | 4 |
| **Hexachlorobencene** | 7 | 0.995 - 0.997 | 50 – 1000 | 1 | 2 |
| **α-HCH** | 16 | 0.994 - 0.99996 | 50 – 1000 | 3 | 5 |
| **β-HCH** | 16 | 0.991 - 0.99997 | 50 – 1000 | 3 | 5 |
| **γ-HCH** | 16 | 0.993 - 0.999999 | 50 – 1000 | 2 | 5 |
| **Isodrin** | 7 | 0.997 - 0.99996 | 50 – 1000 | 2 | 3 |
| **Metolachlor** | 9 | 0.995 - 0.99997 | 50 – 2000 | 4 | 7 |
| **Pentachlorobencenee** | 8 | 0.990 - 0.9997 | 50 – 1000 | 4 | 13 |
| **PCB-28** | 8 | 0.994 - 0.9998 | 50 – 1000 | 2 | 15 |
| **PCB-52** | 8 | 0.994 - 0.99991 | 50 – 1000 | 2 | 9 |
| **PCB-101** | 8 | 0.997 - 0.99993 | 50 – 1000 | 2 | 12 |
| **PCB-118** | 8 | 0.993 - 0.99995 | 50 – 1000 | 3 | 22 |
| **PCB-138** | 8 | 0.992 - 0.99991 | 50 – 1000 | 3 | 19 |
| **PCB-153** | 8 | 0.991 - 0.99998 | 50 – 1000 | 3 | 19 |
| **PCB-180** | 8 | 0.993 - 0.99998 | 50 – 1000 | 4 | 21 |
| **Simazine** | 9 | 0.992 - 0.9997 | 50 – 2000 | 6 | 13 |
| **Terbutylazine** | 9 | 0.997 - 0.9997 | 50 – 2000 | 4 | 7 |

Table S4: Accuracy evaluated by the analysis of two certified reference materials

|  | Water (WatRTM 713) | | Sediment (CNS391) | |
| --- | --- | --- | --- | --- |
| Compound | **Acceptance limits**  **(µg/L)** | **Value measured**  **(µg/L) n=2** | **Certified value**  **(ng/g)** | **Value measured**  **(ng/g) n=4** |
| **Aldrin** | 0.82 – 2.32 | 1.17 ± 0.04 | - | - |
| **Atrazine** | 1.92 – 5.08 | 2.86 ± 0.14 | - | - |
| **Dieldrin** | 0.86 – 2.28 | 1.34 ± 0.04 | - | - |
| **o-p’-DDD** | - | - | 15.5 | 14±2.3 |
| **p-p’-DDD** | - | - | 13.9 | 12±2.0 |
| **o-p’-DDE** | - | - | 39.5 | 31±5.8 |
| **p-p’-DDE** | - | - | 18.8 | 17 ±2.5 |
| **o-p’-DDT** | - | - | 43 | 24 ± 5.25 |
| **p-p’-DDT** | - | - | 10.2 | 11 ± 0.80 |
| **Endrin** | 2.27 – 2.95 | 2.87 ± 0.11 | - | - |
| **Hexachlorobencene** | 1.41 – 3.50 | 1.72 ± 0.11 | 34.5 | 35 ± 3.3 |
| **α-HCH** | - | - | 37.1 | 26 ± 2.6 |
| **β-HCH** | - | - | 21.1 | 25 ± 1.0 |
| **γ-HCH** | 1.18 – 3.10 | 2.07 ± 0.02 | 9.5 | 12 ± 1.0 |
| **Metolachlor** | 5.61 – 14.8 | 7.41 ± 0.22 | - | - |
| **PCB-28** | - | - | 44.9 | 41 ± 3.1 |
| **PCB-52** | - | - | 64.6 | 67 ± 11 |
| **PCB-101** | - | - | 45.7 | 44 ± 9.3 |
| **PCB-118** | - | - | 24 | 28 ± 5.1 |
| **PCB-138** | - | - | 34.6 | 34 ±7.8 |
| **PCB-153** | - | - | 50.1 | 34±5.2 |
| **PCB-180** | - | - | 54.7 | 41±5.2 |
| **PCB-28** | - | - | 44.9 | 41 ± 3.1 |
| **Simazine** | 1.99 – 5.23 | 2.31 ± 0.07 | - | - |


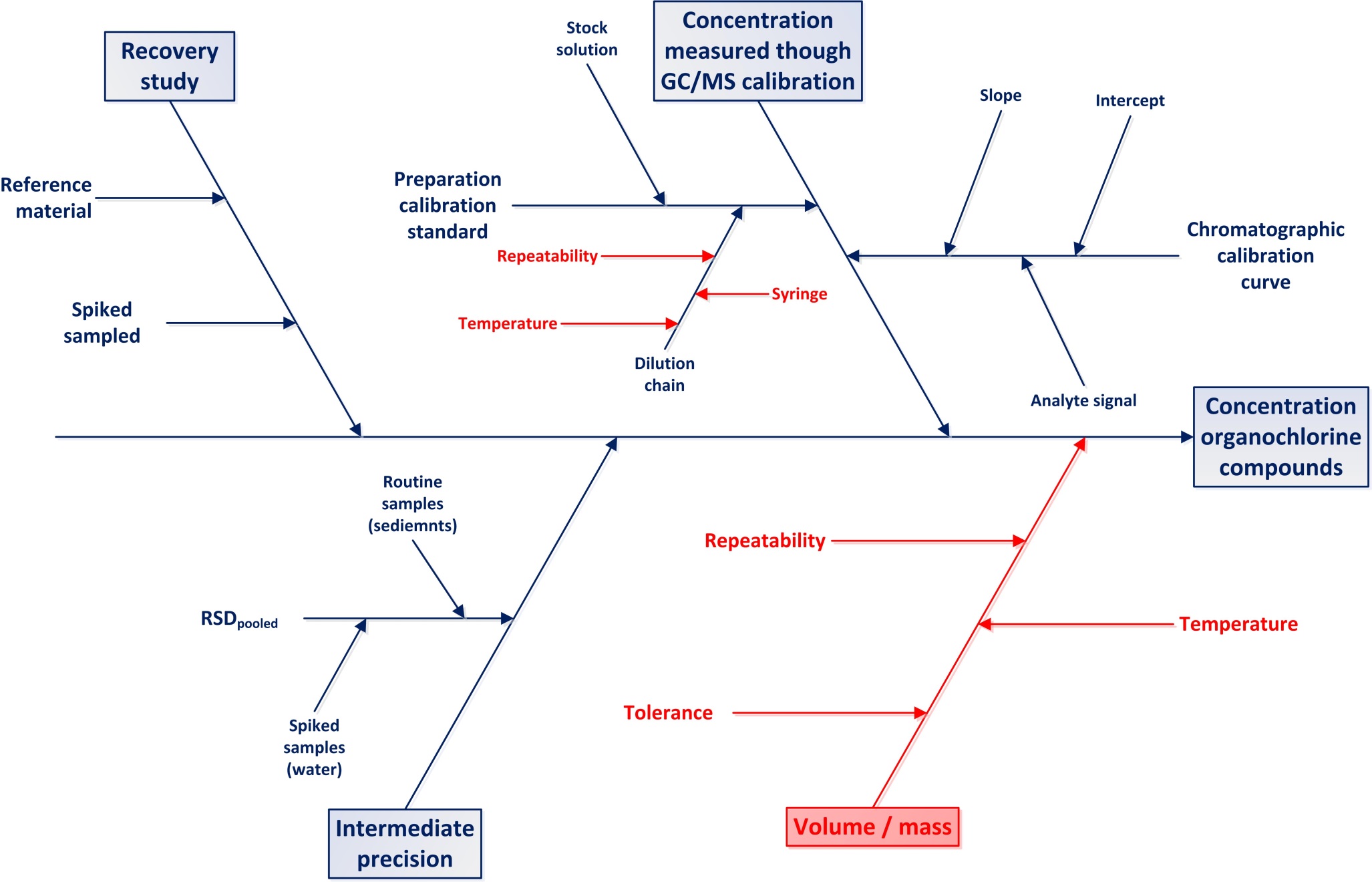


Fig. S3: Cause and effect diagram


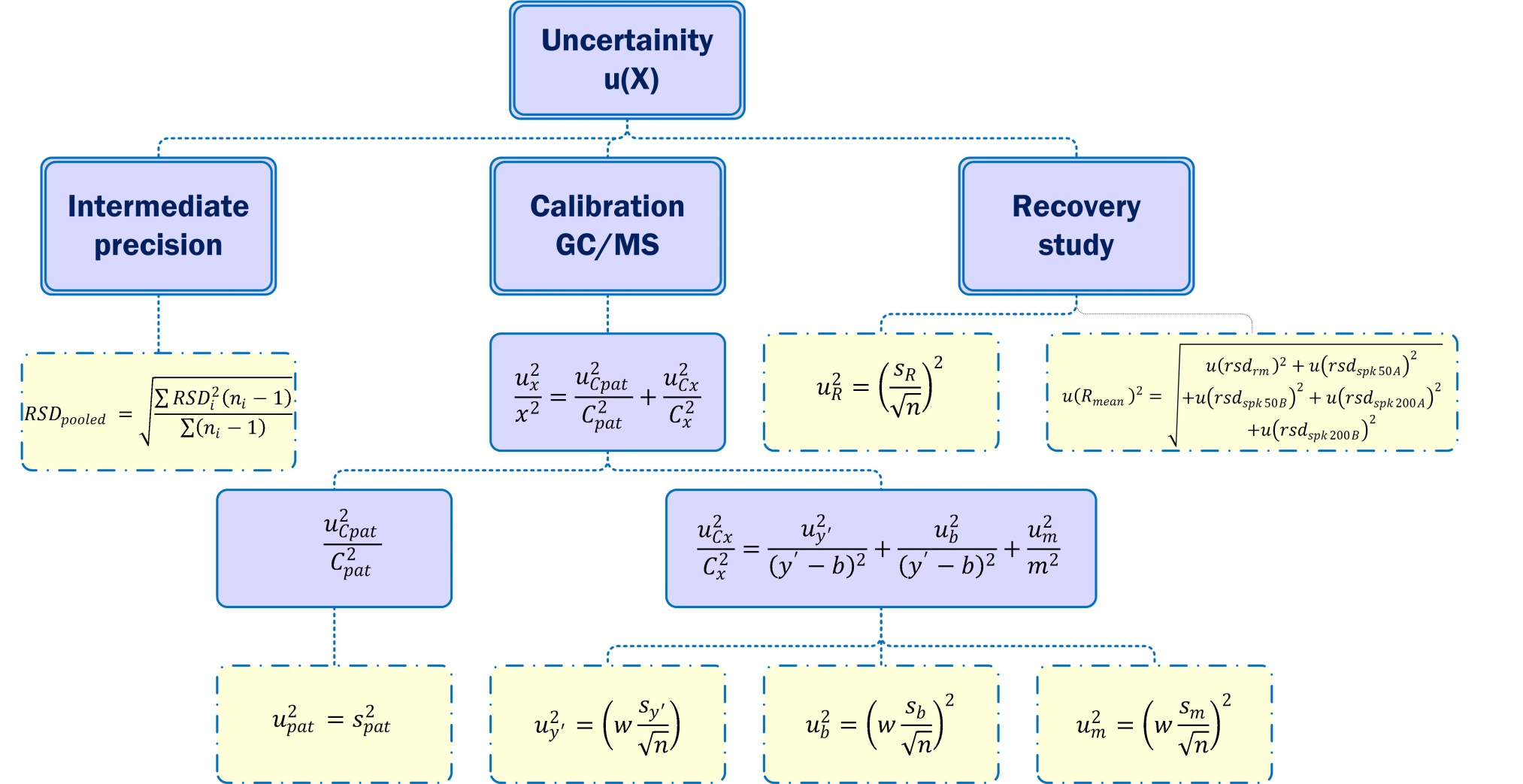


Fig. S4: Equations to calculate uncertainty

Table S5: Uncertainty from analytical signal (u_y_^2^)

| Compound | y’ | s_y_ | n | u_y_^2^ |
| --- | --- | --- | --- | --- |
| **Aldrin** | 0.006 | 0.001 | 11 | 8.9 10^-7^ |
| **Atrazine** | 0.011 | 0.0012 | 18 | 1.0 10^-6^ |
| **Dieldrin** | 0.012 | 0.0046 | 17 | 2.0 10^-5^ |
| **o-p’-DDD** | 0.027 | 0.0035 | 13 | 1.2 10^-5^ |
| **p-p’-DDD** | 0.027 | 0.0068 | 14 | 3.4 10^-5^ |
| **o-p’-DDE** | 0.03 | 0.0029 | 21 | 4.8 10^-6^ |
| **p-p’-DDE** | 0.022 | 0.0027 | 20 | 5.1 10^-5^ |
| **o-p’-DDT** | 0.023 | 0.0032 | 11 | 9.9 10^-6^ |
| **p-p’-DDT** | 0.024 | 0.0029 | 11 | 1.2 10^-5^ |
| **Endrin** | 0.053 | 0.0253 | 12 | 6.20 10^-4^ |
| **Hexachlorobencene** | 0.02 | 0.0032 | 13 | 9.60 10^-6^ |
| **Isodrin** | 0.012 | 0.0028 | 11 | 1.20 10^-5^ |
| **α-HCH** | 0.006 | 0.0009 | 15 | 8.10 10^-7^ |
| **β-HCH** | 0.005 | 0.0005 | 15 | 2.20 10^-7^ |
| **γ-HCH** | 0.005 | 0.0007 | 15 | 4.10 10^-7^ |
| **Metolachlor** | 0.024 | 0.004 | 14 | 1.60 10^-5^ |
| **PCB-28** | 0.02 | 0.0035 | 13 | 1.20 10^-5^ |
| **PCB-52** | 0.018 | 0.0037 | 13 | 1.30 10^-5^ |
| **PCB-101** | 0.04 | 0.0114 | 13 | 1.20 10^-4^ |
| **PCB-118** | 0.053 | 0.0154 | 13 | 2.30 10^-4^ |
| **PCB-138** | 0.044 | 0.0172 | 15 | 2.80 10^-4^ |
| **PCB-153** | 0.045 | 0.0107 | 15 | 1.10 10^-4^ |
| **PCB-180** | 0.032 | 0.0042 | 16 | 1.70 10^-5^ |
| **Pentachlorobencene** | 0.017 | 0.004 | 13 | 2.80 10^-5^ |
| **Simazine** | 0.008 | 0.0023 | 17 | 4.90 10^-6^ |
| **Terbuthylazine** | 0.018 | 0.0022 | 18 | 3.60 10^-6^ |

Table S6: Uncertainty from slope (u_m_^2^) and y-intercept (u_b_^2^)

| Compound | b | m | s_b_ | s_m_ | u_b_^2^ | u_m_^2^ |
| --- | --- | --- | --- | --- | --- | --- |
| **Aldrin** | -0.003 | 1.20 10^-4^ | 0.0038 | 8.30 10^-6^ | 1.60 10^-6^ | 7.70 10^-12^ |
| **Atrazine** | -0.0051 | 2.70 10^-4^ | 0.0057 | 1.10 10^-5^ | 2.60 10^-6^ | 9.30 10^-12^ |
| **Dieldrin** | -0.0008 | 2.40 10^-4^ | 0.012 | 2.30 10^-5^ | 1.50 10^-5^ | 5.50 10^-11^ |
| **o-p’-DDD** | -0.0229 | 8.00 10^-4^ | 0.0188 | 3.60 10^-5^ | 3.40 10^-5^ | 1.30 10^-10^ |
| **p-p’-DDD** | -0.0248 | 8.40 10^-4^ | 0.0193 | 3.80 10^-5^ | 3.60 10^-5^ | 1.40 10^-10^ |
| **o-p’-DDE** | -0.0024 | 6.40 10^-4^ | 0.0056 | 1.10 10^-5^ | 2.20 10^-6^ | 8.60 10^-12^ |
| **p-p’-DDE** | -0.0028 | 5.20 10^-4^ | 0.0051 | 1.00 10^-5^ | 1.80 10^-6^ | 6.90 10^-12^ |
| **o-p’-DDT** | -0.0256 | 7.10 10^-4^ | 0.0193 | 3.80 10^-5^ | 4.90 10^-5^ | 1.90 10^-10^ |
| **p-p’-DDT** | -0.025 | 7.20 10^-4^ | 0.0167 | 3.30 10^-5^ | 3.70 10^-5^ | 1.50 10^-10^ |
| **Endrin** | 0.0032 | 8.50 10^-5^ | 0.0049 | 1.00 10^-5^ | 2.90 10^-6^ | 1.20 10^-11^ |
| **Hexachlorobencene** | -0.0007 | 3.40 10^-4^ | 0.0038 | 8.20 10^-6^ | 1.60 10^-6^ | 7.50 10^-12^ |
| **Isodrin** | -0.0067 | 1.80 10^-4^ | 0.0103 | 2.00 10^-5^ | 9.50 10^-6^ | 3.50 10^-11^ |
| **α-HCH** | -0.0041 | 1.40 10^-4^ | 0.0033 | 5.90 10^-6^ | 9.80 10^-7^ | 3.10 10^-12^ |
| **β-HCH** | -0.0049 | 1.30 10^-4^ | 0.0037 | 6.50 10^-6^ | 1.20 10^-6^ | 3.90 10^-12^ |
| **γ-HCH** | -0.0046 | 1.30 10^-4^ | 0.0045 | 7.90 10^-6^ | 1.80 10^-6^ | 5.70 10^-12^ |
| **Metolachlor** | -0.0207 | 6.60 10^-4^ | 0.0121 | 2.10 10^-5^ | 1.50 10^-5^ | 4.40 10^-11^ |
| **PCB-28** | -0.003 | 4.00 10^-4^ | 0.0047 | 1.00 10^-5^ | 2.40 10^-6^ | 1.10 10^-11^ |
| **PCB-52** | 0.0004 | 3.20 10^-4^ | 0.0044 | 9.60 10^-6^ | 2.20 10^-6^ | 1.00 10^-11^ |
| **PCB-101** | 0.0026 | 4.10 10^-4^ | 0.0067 | 1.40 10^-5^ | 5.00 10^-6^ | 2.30 10^-11^ |
| **PCB-118** | 0.0018 | 5.70 10^-4^ | 0.0097 | 2.10 10^-5^ | 1.00 10^-5^ | 4.80 10^-11^ |
| **PCB-138** | 0.001 | 4.80 10^-4^ | 0.0111 | 2.10 10^-5^ | 1.20 10^-5^ | 4.40 10^-11^ |
| **PCB-153** | -0.0021 | 5.30 10^-4^ | 0.0108 | 2.10 10^-5^ | 8.90 10^-6^ | 3.30 10^-11^ |
| **PCB-180** | 0.0007 | 4.90 10^-4^ | 0.0117 | 2.50 10^-5^ | 1.50 10^-5^ | 7.10 10^-11^ |
| **Pentachlorobencene** | 0.002 | 2.80 10^-4^ | 0.0012 | 2.50 10^-6^ | 1.50 10^-7^ | 6.90 10^-13^ |
| **Simazine** | -0.0048 | 1.90 10^-4^ | 0.0039 | 7.20 10^-6^ | 1.30 10^-6^ | 4.40 10^-12^ |
| **Terbuthylazine** | -0.0073 | 4.20 10^-4^ | 0.0094 | 1.80 10^-5^ | 7.10 10^-6^ | 2.50 10^-11^ |

Table S7: Uncertainty associated to concentration measured through calibration

| Compound | u_y_^2^/(y-b)^2^ | u_b_^2^/(y-b)^2^ | u_m_^2^/m^2^ | u_Cx_^2^/C_x_^2^ |
| --- | --- | --- | --- | --- |
| **Aldrin** | 0.011 | 0.0202 | 1.90 10^-4^ | 0.032 |
| **Atrazine** | 0.004 | 0.0099 | 1.20 10^-4^ | 0.014 |
| **Dieldrin** | 0.121 | 0.0894 | 9.40 10^-4^ | 0.212 |
| **o-p’-DDD** | 0.005 | 0.0137 | 2.00 10^-4^ | 0.019 |
| **p-p’-DDD** | 0.013 | 0.0133 | 2.00 10^-4^ | 0.026 |
| **o-p’-DDE** | 0.005 | 0.0021 | 2.10 10^-5^ | 0.007 |
| **p-p’-DDE** | 0.008 | 0.0029 | 2.60 10^-5^ | 0.011 |
| **o-p’-DDT** | 0.004 | 0.0206 | 3.80 10^-4^ | 0.025 |
| **p-p’-DDT** | 0.005 | 0.0153 | 2.80 10^-4^ | 0.021 |
| **Endrin** | 0.248 | 0.0012 | 1.70 10^-3^ | 0.251 |
| **Hexachlorobencene** | 0.023 | 0.0039 | 6.50 10^-5^ | 0.027 |
| **Isodrin** | 0.033 | 0.0269 | 1.10 10^-3^ | 0.061 |
| **α-HCH** | 0.008 | 0.0098 | 1.60 10^-4^ | 0.018 |
| **β-HCH** | 0.002 | 0.0129 | 2.50 10^-4^ | 0.015 |
| **γ-HCH** | 0.004 | 0.018 | 3.20 10^-4^ | 0.022 |
| **Metolachlor** | 0.008 | 0.0076 | 1.00 10^-4^ | 0.016 |
| **PCB-28** | 0.022 | 0.0045 | 7.10 10^-5^ | 0.027 |
| **PCB-52** | 0.43 | 0.0071 | 9.70 10^-5^ | 0.05 |
| **PCB-101** | 0.089 | 0.0036 | 1.40 10^-4^ | 0.093 |
| **PCB-118** | 0.086 | 0.0039 | 1.50 10^-4^ | 0.09 |
| **PCB-138** | 0.152 | 0.0063 | 1.90 10^-4^ | 0.158 |
| **PCB-153** | 0.049 | 0.004 | 1.20 10^-4^ | 0.053 |
| **PCB-180** | 0.017 | 0.0156 | 2.90 10^-4^ | 0.033 |
| **Pentachlorobencene** | 0.119 | 0.0006 | 8.70 10^-6^ | 0.12 |
| **Simazine** | 0.032 | 0.0085 | 1.20 10^-4^ | 0.04 |
| **Terbuthylazine** | 0.006 | 0.0115 | 1.40 10^-4^ | 0.017 |

Table S8: Uncertainty associated to recovery of selected pesticides in water samples

| Compound | Recovery | s | n | u_rec_ | u_rec_/R | u_rec_/R (%) |
| --- | --- | --- | --- | --- | --- | --- |
| **Aldrin** | 0.85 | 0.040 | 6 | 0.016 | 0.019 | 2 |
| **Atrazine** | 1.11 | 0.141 | 12 | 0.041 | 0.037 | 4 |
| **o-p’-DDD** | 0.55 | 0.134 | 12 | 0.039 | 0.070 | 7 |
| **p-p’-DDD** | 0.72 | 0.220 | 18 | 0.052 | 0.072 | 7 |
| **o-p’-DDE** | 0.37 | 0.079 | 12 | 0.023 | 0.062 | 6 |
| **p-p’-DDE** | 0.44 | 0.151 | 16 | 0.038 | 0.086 | 9 |
| **o-p’-DDT** | 0.40 | 0.082 | 8 | 0.029 | 0.073 | 7 |
| **p-p’-DDT** | 0.65 | 0.256 | 12 | 0.074 | 0.114 | 11 |
| **Dieldrin** | 0.82 | 0.202 | 18 | 0.048 | 0.058 | 6 |
| **Endrin** | 0.81 | 0.443 | 18 | 0.104 | 0.130 | 13 |
| **Hexachlorobencene** | 0.93 | 0.149 | 6 | 0.061 | 0.066 | 7 |
| **α-HCH** | 1.28 | 0.338 | 18 | 0.080 | 0.062 | 6 |
| **β-HCH** | 1.17 | 0.254 | 18 | 0.060 | 0.051 | 5 |
| **γ-HCH** | 1.20 | 0.273 | 18 | 0.064 | 0.054 | 5 |
| **Isodrin** | 0.88 | 0.071 | 6 | 0.029 | 0.033 | 3 |
| **Metolachlor** | 0.98 | 0.120 | 12 | 0.035 | 0.035 | 4 |
| **Simazine** | 1.17 | 0.181 | 12 | 0.052 | 0.044 | 4 |
| **Terbutylazine** | 0.91 | 0.188 | 12 | 0.054 | 0.060 | 6 |

Table S9: Uncertainty associated to recovery of selected pesticides in sediment samples

| Compound | u(rsd)_rm_ | u(rsd)_spk50A_ | u(rsd)_spk50B_ | u(rsd)_spk200A_ | u(rsd)_spk200B_ | u(R_mean_) |
| --- | --- | --- | --- | --- | --- | --- |
| **p-p’-DDD** | 0.083 |  | 0.021 |  | 0.099 | 0.097 |
| **p-p’-DDE** | 0.073 | 0.003 | 0.001 | 0.001 | 0.005 | 0.073 |
| **p-p’-DDT** | 0.036 | 0.001 | 0.001 |  |  | 0.036 |
| **Hexachlorobencene** | 0.047 | 0.124 | 0.143 | 0.050 | 0.043 | 0.205 |
| **α-HCH** | 0.049 | 0.062 | 0.012 | 0.037 | 0.017 | 0.090 |
| **β-HCH** | 0.019 | 0.044 | 0.037 | 0.043 | 0.033 | 0.081 |
| **γ-HCH** | 0.042 | 0.007 | 0.038 | 0.030 | 0.006 | 0.065 |
| **PCB-28** | 0.038 | 0.009 | 0.024 | 0.011 | 0.011 | 0.049 |
| **PCB-52** | 0.080 | 0.018 | 0.042 | 0.041 | 0.116 | 0.154 |
| **PCB-101** | 0.106 | 0.008 | 0.020 | 0.019 | 0.053 | 0.122 |
| **PCB-118** | 0.092 | 0.008 | 0.023 | 0.025 | 0.031 | 0.103 |
| **PCB-138** | 0.113 | 0.028 | 0.026 | 0.017 | 0.012 | 0.121 |
| **PCB-153** | 0.076 | 0.010 | 0.033 | 0.008 | 0.008 | 0.084 |
| **PCB-180** | 0.063 | 0.005 | 0.026 | 0.013 | 0.011 | 0.071 |

Table S10: Uncertainty associated to intermediate precision for the analysis of selected compounds in water samples

|  | Recovery study (1000 ng/L) | | | | | | Recovery study (500 ng/L) | | | | | | Recovery study (250 ng/L) | | | | | |  |  |
| --- | --- | --- | --- | --- | --- | --- | --- | --- | --- | --- | --- | --- | --- | --- | --- | --- | --- | --- | --- | --- |
|  | **Recovery A** | | **Recovery B** | | **Recovery C** | | **Recovery A** | | **Recovery B** | | **Recovery C** | | **Recovery A** | | **Recovery B** | | **Recovery C** | |  |  |
|  | **Avge** | **s** | **Avge** | **s** | **Avge** | **s** | **Avge** | **s** | **Avge** | **s** | **Avge** | **s** | **Avge** | **s** | **Avge** | **s** | **Avge** | **s** | **n** | **rsd _pooled_** |
| **Aldrin** |  |  |  |  |  |  | 0.85 | 0.04 | 0.36 | 0.10 | 0.27 | 0.04 |  |  |  |  |  |  | 3 | 0.082 |
| **Atrazine** | 1.09 | 0.06 | 1.17 | 0.07 | 0.89 | 0.14 |  |  |  |  |  |  | 1.07 | 0.04 | 1.25 | 0.15 | 1.20 | 0.03 | 6 | 0.104 |
| **op’-DDD** | 0.30 | 0.01 | 0.33 | 0.03 | 0.34 | 0.06 |  |  |  |  |  |  | 0.46 | 0.13 | 0.39 | 0.04 | 0.39 | 0.12 | 6 | 0.085 |
| **pp’-DDD** | 0.31 | 0.01 | 0.34 | 0.02 | 0.35 | 0.08 | 0.73 | 0.17 | 0.44 | 0.10 | 1.02 | 0.02 | 0.52 | 0.11 | 0.37 | 0.07 | 0.47 | 0.10 | 9 | 0.096 |
| **op’-DDE** | 0.34 | 0.01 | 0.61 | 0.05 | 0.54 | 0.14 | 1.06 | 0.27 | 0.95 | 0.27 | 0.65 | 0.15 | 0.63 | 0.13 | 0.54 | 0.09 | 0.64 | 0.15 | 9 | 0.172 |
| **pp’-DDE** | 0.57 | 0.02 | 0.63 | 0.06 | 0.52 | 0.19 |  |  |  |  |  |  | 0.77 | 0.22 | 0.57 | 0.12 | 0.74 | 0.10 | 6 | 0.150 |
| **op’-DDT** | 0.30 | 0.02 | 0.43 | 0.04 | 1.00 | 0.00 |  |  |  |  |  |  | 0.43 | 0.10 | 0.44 | 0.09 | 1.00 | 0.00 | 6 | 0.063 |
| **pp’-DDT** | 0.37 | 0.04 | 0.64 | 0.12 | 1.00 | 0.00 | 1.03 | 0.34 | 0.47 | 0.21 | 1.00 | 0.00 | 0.66 | 0.09 | 0.71 | 0.14 | 1.00 | 0.00 | 9 | 0.160 |
| **Dieldrin** | 0.73 | 0.02 | 0.91 | 0.07 | 0.83 | 0.49 | 1.46 | 0.19 | 1.20 | 0.24 | 0.31 | 0.06 | 0.70 | 0.11 | 0.06 | 0.01 | 1.06 | 0.16 | 9 | 0.217 |
| **Endrin** | 0.68 | 0.01 | 0.82 | 0.02 | 0.70 | 0.08 | 1.18 | 0.17 | 1.10 | 0.18 | 0.74 | 0.08 | 0.69 | 0.11 | 0.67 | 0.07 | 0.83 | 0.15 | 9 | 0.120 |
| **HCB** |  |  |  |  |  |  | 1.04 | 0.11 | 0.81 | 0.03 | 0.48 | 0.05 |  |  |  |  |  |  | 3 | 0.090 |
| **α-HCH** | 1.26 | 0.11 | 1.33 | 0.25 | 0.98 | 0.01 | 1.92 | 0.24 | 1.61 | 0.11 | 1.00 | 0.08 | 1.24 | 0.39 | 1.24 | 0.10 | 0.93 | 0.04 | 9 | 0.199 |
| **β-HCH** | 1.25 | 0.07 | 1.13 | 0.09 | 0.83 | 0.31 | 1.51 | 0.11 | 1.53 | 0.28 | 1.07 | 0.02 | 1.06 | 0.18 | 1.08 | 0.18 | 1.05 | 0.01 | 9 | 0.182 |
| **γ-HCH** | 1.25 | 0.07 | 1.23 | 0.18 | 0.87 | 0.21 | 1.68 | 0.02 | 1.39 | 0.19 | 0.98 | 0.06 | 1.00 | 0.35 | 1.34 | 0.03 | 1.03 | 0.01 | 9 | 0.176 |
| **Isodrin** |  |  |  |  |  |  | 0.88 | 0.07 | 0.43 | 0.16 | 0.31 | 0.01 |  |  |  |  |  |  | 3 | 0.121 |
| **Methlor** | 1.12 | 0.01 | 1.11 | 0.01 | 0.94 | 0.09 |  |  |  |  |  |  | 0.90 | 0.12 | 1.01 | 0.08 | 0.84 | 0.03 | 6 | 0.076 |
| **Simazine** | 1.43 | 0.09 | 1.27 | 0.08 | 0.97 | 0.19 |  |  |  |  |  |  | 1.05 | 0.00 | 1.24 | 0.15 | 1.09 | 0.01 | 6 | 0.121 |
| **Terbuty** | 1.12 | 0.02 | 1.13 | 0.04 | 0.90 | 0.09 |  |  |  |  |  |  | 0.73 | 0.14 | 0.84 | 0.12 | 0.71 | 0.03 | 6 | 0.095 |

Table S11: Variance from the analysis of each sediment by duplicate.

| Sample | PClB | α-HCH | β-HCH | γ-HCH | HCB | op’-DDE | pp’-DDE | op’-DDD | pp’-DDD | op’-DDT | pp’-DDT | PCB 28 | PCB 52 | PCB 101 | PCB 118 | PCB 153 | PCB 138 | PCB 180 |
| --- | --- | --- | --- | --- | --- | --- | --- | --- | --- | --- | --- | --- | --- | --- | --- | --- | --- | --- |
| **1** | 0.366 | 0.019 | 0.222 | 0.003 | 0.006 | 0.012 | 0.037 | 0.009 | 0.076 |  |  | 0.003 | 0.01 | 0.125 | 0.015 | 0.002 |  |  |
| **2** | 0.698 | 0.025 | 0.002 | 0.009 | 0.061 | 0.099 | 0.005 | 0.007 |  |  |  |  | 0.002 | 0.003 |  | 0.017 |  |  |
| **3** | 0.055 | 0.035 |  | 0.022 | 0.02 | 0.031 | 0.021 | 0.016 | 0.027 |  |  | 0.019 | 0.028 | 0.029 |  | 0.025 |  |  |
| **4** |  | 1.037 | 0.155 |  | 0.955 | 0.08 | 0.002 | 0.038 | 0.039 |  |  | 0.242 | 0.16 | 0.13 | 0.13 | 0.278 |  |  |
| **5** | 0.015 | 0.004 |  | 0.008 | 0.25 | 0.002 |  | 0.003 | 0.014 |  |  |  | 0.002 | 0.031 | 0.068 | 0.01 |  |  |
| **6** | 0.007 | 0.005 |  | 0.001 |  | 0.008 |  | 0.002 | 0.073 |  |  |  | 0.001 | 0.001 | 0.016 | 0.004 |  |  |
| **7** | 0.014 | 0.03 | 0.003 | 0.004 |  | 0.003 | 0.004 | 0.038 | 0.01 |  |  | 0.001 | 0.001 | 0.003 | 0.002 | 0.004 |  |  |
| **8** | 0.255 | 0.048 | 0.015 | 0.022 | 0.082 | 0.099 | 0.031 | 0.028 | 0.278 |  |  | 0.129 | 0.056 | 0.06 |  | 0.037 |  |  |
| **9** | 0.039 | 0.005 | 0.324 | 0.023 | 0.005 | 0.012 | 0.028 | 0.162 | 0.718 |  |  | 0.008 | 0.054 | 0.12 | 0.281 | 0.116 |  |  |
| **10** | 1.115 | 0.173 | 0.017 | 0.007 | 0.102 | 0.004 |  | 0.045 | 0.332 |  |  | 0.001 | 0.001 | 0.001 |  | 0.001 |  |  |
| **11** | 0.044 | 0.005 | 0.016 | 0.002 | 0.024 | 0.022 | 0.062 | 0.198 | 1.414 |  |  | 0.241 | 0.371 | 0.494 |  |  |  |  |
| **12** |  | 0.056 | 0.019 | 0.044 | 0.209 | 0.02 | 0.002 | 0.08 | 0.047 |  |  | 0.056 | 0.132 | 0.112 | 0.051 | 0.082 | 0.146 | 0.048 |
| **13** |  | 0.009 | 0.074 | 0.004 | 0.001 | 0.115 | 0.112 | 0.082 | 0.052 | 0.082 | 0.002 | 0.019 | 0.06 | 0.086 | 0.08 | 0.128 | 0.092 | 0.12 |
| **14** | 0.038 |  | 0.039 |  | 0.055 | 0.137 | 0.181 | 0.001 |  | 0.5 |  | 0.097 | 0.001 | 0.052 | 0.199 | 0.308 | 0.175 | 0.728 |
| **15** | 0.316 | 0.004 | 0.002 | 0.012 |  | 0.139 | 0.074 | 0.047 | 0.119 |  |  | 0.01 | 0.007 | 0.011 |  | 0.01 |  |  |
| **16** |  | 0.009 | 0.199 |  | 0.062 | 0.25 | 0.874 | 0.135 | 0.058 |  |  |  | 0.036 |  |  |  |  |  |
| **17** | 0.182 | 0.165 | 0.915 |  | 0.107 |  | 0.006 | 0.018 | 0.023 |  | 0.033 | 0.005 |  | 0.002 | 0.017 | 0.422 | 0.513 | 0.012 |
| **18** | 0.029 | 0.008 | 0.024 | 0.036 | 0.087 | 0.032 | 0.02 | 0.214 | 0.129 | 0.017 | 0.355 | 0.076 | 0.095 | 0.064 | 0.011 | 0.024 | 0.022 | 0.031 |
| **19** | 0.008 | 0.013 | 0.087 | 0.018 | 0.002 | 0.001 | 0.002 | 0.004 | 0.03 | 0.167 | 0.488 | 0.002 | 0.001 | 0.112 | 0.001 | 0.005 |  |  |
| **20** | 0.005 | 0.009 | 0.006 | 0.031 | 0.018 | 0.007 | 0.002 |  | 0.045 | 0.641 | 1.143 | 0.005 | 0.003 | 0.001 | 0.019 | 0.32 | 0.49 |  |
| **21** | 1.01 | 0.014 | 0.015 |  | 0.476 | 0.08 | 0.017 | 0.036 | 0.008 | 0.041 |  | 0.407 | 0.287 |  |  |  |  |  |
| **22** | 0.387 | 0.022 | 0.01 | 0.028 |  | 0.017 | 0.001 | 0.031 | 0.039 | 0.056 |  | 0.004 | 0.006 | 0.008 | 0.003 | 0.002 | 0.003 |  |
| **23** | 0.043 | 0.032 | 0.005 | 0.037 |  | 0.065 | 0.016 | 0.045 | 0.133 | 0.188 |  | 0.056 | 0.013 | 0.019 |  | 0.031 |  |  |
| **24** | 0.009 | 0.038 |  | 0.017 | 0.144 | 0.183 | 0.08 | 0.062 | 0.018 |  |  | 0.14 | 0.171 | 0.325 |  | 0.81 |  |  |
| **25** | 0.004 | 0.028 | 1.284 | 0.01 | 0.066 | 0.004 | 0.005 |  | 0.018 | 0.005 | 0.628 | 0.028 | 0.018 | 0.01 | 0.012 | 0.002 |  |  |
| **26** | 0.017 | 0.005 | 0.001 | 0.006 | 0.014 | 0.015 | 0.023 | 0.01 | 0.011 |  |  | 0.183 | 0.09 | 0.133 |  | 0.133 |  |  |
| **27** |  | 0.663 | 0.122 | 0.763 |  | 0.031 | 0.015 | 0.009 | 0.003 |  |  | 0.191 | 0.025 | 0.006 |  | 0.004 |  |  |
| **28** | 0.012 | 0.001 |  | 0.374 | 0.593 | 0.034 | 0.06 | 0.138 | 0.185 |  |  | 0.04 | 0.02 | 0.098 |  | 0.101 |  |  |
| **29** | 0.016 | 0.015 | 0.02 |  | 0.195 |  |  |  | 0.088 | 0.012 |  | 0.056 | 0.004 |  |  | 0.015 | 0.001 | 0.001 |
| **30** | 0.118 | 0.036 | 0.056 | 0.001 | 0.02 | 0.001 | 0.003 | 0.006 | 0.007 | 0.009 | 0.335 | 0.04 | 0.023 |  | 0.006 | 0.034 | 0.015 | 0.018 |
| **31** |  | 0.118 |  | 0.047 | 0.022 | 0.013 | 0.01 | 0.012 | 0.025 | 0.212 |  | 0.019 | 0.023 | 0.025 | 0.046 | 0.019 | 0.032 | 0.019 |
| **32** | 0.26 | 0.101 | 0.038 | 0.187 | 0.52 | 0.036 | 0.046 | 0.139 | 0.08 | 0.18 |  | 0.159 | 0.111 | 0.07 |  | 0.044 |  |  |
| **33** | 0.026 | 0.336 | 0.065 | 0.019 | 0.524 | 0.114 | 0.021 | 0.052 | 0.103 | 1.997 | 0.966 | 0.004 | 0.001 | 0.634 | 0.062 | 0.031 | 0.025 |  |
